# Oncogenic p53 induces mitotic errors in lung cancer cells by recopying DNA replication forks conferring targetable proliferation advantage

**DOI:** 10.1038/s41418-026-01670-4

**Published:** 2026-01-26

**Authors:** Shilpa Singh, Lilia Gheghiani, Brandon Velasco, Rebecca Frum, Steven R. Grossman, Brad Windle, Sumitra Deb, Swati Palit Deb

**Affiliations:** 1 VCU Massey Cancer Center, Virginia Commonwealth University, Richmond, VA USA; 2 Department of Cellular Molecular Genetic Medicine, Virginia Commonwealth University, Richmond, VA USA; 3 Department of Internal Medicine, Division of Hematology, Oncology and Palliative Care, Virginia Commonwealth University, Richmond, VA USA; 4 Department of Oral and Craniofacial Molecular Biology, Philips Institute for Oral Health Research, VCU School of Dentistry, Virginia Commonwealth University, Richmond, VA USA

**Keywords:** Oncogenes, Cancer genetics

## Abstract

Mutations in tumor suppressor p53 that gain oncogenic functions (Onc-p53) are frequent in lungs and many other solid tumors often associated with chromosome aberrations. Why cells or tumors with Onc-p53 develop chromosomal aberrations and whether the abnormalities contribute to tumor growth remain elusive. Evidence in this communication demonstrate for the first time that replication stress induced by Onc-p53 triggers re-copying of DNA replication forks, which generates replication intermediates that cause persistent mitotic aberration and DNA segregation errors. Replication intermediates from re-copied replication forks induced by Onc-p53 activate ATM signaling, which stabilizes Onc-p53, reinforces its ability to upregulate replication factors for sustaining replication stress, thus generating a feedforward cycle accelerating tumor formation. In agreement with this observation our time lapse video microscopy show in real time that persistent mitotic aberration and DNA segregation errors induced by Onc-p53 confer selective growth advantage. Accordingly, human lung tumors with Onc-p53 show selection of cells with mitotic aberration during serial passages. Knock down of active replication forks reduces re-copied fork generation by Onc-p53 and specifically induces apoptotic death of lung cancer cells expressing Onc-p53 in xenograft lung tumors in cooperation with inhibitors of ATM activation, deselecting cells with Onc-p53 with mitotic errors. This communication reveals a novel mechanism which interconnects replication stress induced by Onc-p53 to its stabilization and ability to generate chromosomal aberration in lung cancer cells that both accelerate tumor growth and serve as a targetable therapeutic vulnerability. These findings will be extremely valuable for tumor-specific treatment of a high percentage of cancer patients with p53 mutation.

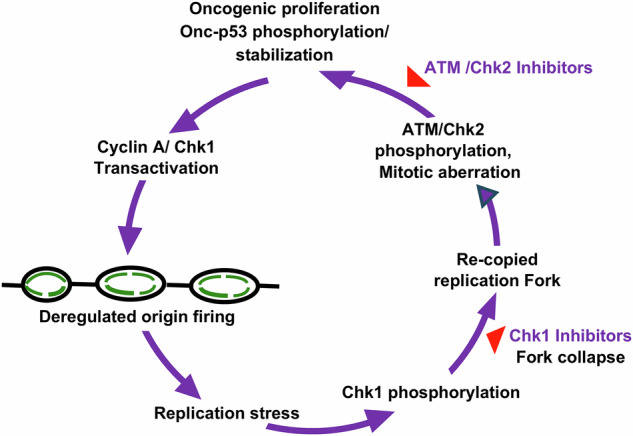

## Introduction

Tumor suppressor p53 mutation is frequent in all types of lung cancers [1]. While oncogenic mutations of wild-type (WT) p53 cause loss of tumor suppressor functions [2], a majority of the mutations are missense mutations, which also show gain of oncogenic properties, often termed GOF p53 (Onc-p53) [3–8]. Depletion or destabilization of endogenous Onc-p53 in human lung cancer cells inhibits tumor growth indicating that Onc-p53 mutations establish dependency in lung cancer cells for tumor formation [9–13]. While the acquired biochemical functions and tumor promoting abilities of Onc-p53 have been reported widely, how its biochemical activities generate chromosomal aberration and establish dependency for persistent intractable tumor growth is not understood.

While deregulated DNA synthesis has been a striking feature of oncogenesis, the mechanism or significance of this observation remains underexplored. In contrast to WT p53, which controls S phase entry of lung cells, p53-null or p53-depleted lung cells, or cells with Onc-p53, show unrestricted S phase entry [2]. However, only lung cells with Onc-p53 show a robust increase in the frequency of DNA replication origin firing, which allows rapid genome doubling and hastened mitotic entry [14]. Onc-p53 transcriptionally activates expression of a wide array of genes that are crucial for its oncogenic function [8, 14–17]. This transcriptional upregulation and expression of replication factors driven by Onc-p53 is required for its ability not only to increase the frequency of origin firing, but also to induce tumor formation [14, 18].

Increased frequency of replication origin firing by oncogenes is known to generate replication stress [19], which induces replication fork collapse due to deprivation of replication factors or nucleotides needed for replication fork progression. Thus, development of replication stress often causes replicative senescence creating a barrier for oncogenic transformation [20–22]. In contrast, Onc-p53 has the unique capability of upregulating the expression of replication factors that prevent replication fork collapse [16, 23]. Onc-p53, however, may still generate replication stress due to topological stress caused by increased origin firing [14]. We wished to investigate how cancer cells process Onc-p53-induced replication forks during cell proliferation

For accurate propagation of an equal number of chromosomes in daughter cells, DNA replication and chromosome segregation are controlled by checkpoint pathways, whereas tumors or cancer cells often evade these controls and proliferate with chromosome segregation errors [24]. This communication reports that increases in the frequency of origin firing by Onc-p53 activates markers of replication stress, which leads to recopying of once replicated DNA generating unresolved DNA fragments. In agreement with reports, that unresolved DNA fragments often interfere with chromosome alignment and processing during anaphase segregation [25–27], human lung cancer cells and tumors displayed Onc-p53-induced chromosome segregation errors while DNA fragments generated by the recopied replication forks activated ATM signaling which stabilized Onc-p53 creating a self-sustaining feedforward loop and selective proliferation of tumors with segregation errors. Release of replication stress specifically induced apoptotic death of lung cancer cells with Onc-p53, demonstrating their dependency on replication stress induced by Onc-p53 for survival and tumor growth.

## Results

### Onc-p53 activates markers of DNA replication stress in lung cancer cells

**Fig. 1:**
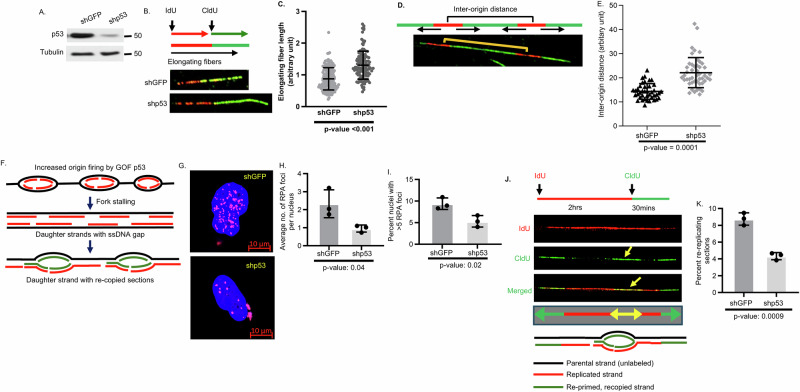
Onc-p53 activates markers of replication stress showing shortened track length of replicating fibers and inter-origin distances, increased chromatin loading of RPA and re-copying of once replicated DNA in a single S phase during DNA replication of lung cancer cells. **A** Immunoblot analysis to show Onc-p53 levels in mock-depleted (shGFP) and p53-depleted (shp53) H1975 lung cancer cells. **B** Fiber analysis of replicating DNA undergoing elongation in partially synchronized shGFP or shp53 H1975 lung cancer cells in early S phase. The scheme of sequential labeling with IdU (red fluorescence) and CldU (green fluorescence) is shown in the upper panel along with representative fiber images (Magnification ×63). **C** Swarm plot analysis indicates comparison of track lengths of 150 to 200 elongating DNA fibers. **D** Distance between contiguous bi-directional origins detected by fiber analysis. Representative fiber image shown (Magnification ×63). **E** Swarm plot analysis indicates inter-origin distances between approximately 40 contiguous bi-directional origins from each sample. **F** Scheme explaining single strand gap generation and recopying of once replicated DNA by Onc-p53. **G** Representative confocal images (Magnification ×40) of nuclei with RPA foci in shGFP and shp53 H1975 cells. Bar graphs comparing (**H**) average number of RPA foci per nuclei and (**I**) percent of nuclei with more than 5 foci. **J** Confocal image of a representative replicating DNA fiber sequentially pulse labeled with IdU (red) and CldU (green) as described (upper panel, **J**) shown in red, green, and merged channels. Re-copied forks (indicated by arrow) were detected by elongating green fibers labeled with second analog, CldU, copying the newly replicated red fibers labeled with the first analog, IdU, generating yellow fiber in the merged image. The scheme (bottom panel) shows re-copying (green) of once replicated DNA (red). **K** The bar graph compares the percentage of re-copied forks in shGFP and shp53 H1975 cells. The percentage indicated represents the fraction of elongating green fibers copying once replicated red fibers (generating yellow fibers) in a single S phase. *p* values are shown.

Since Onc-p53 increases the frequency of DNA replication origin firing in lung cancer cells [14], the development of replication stress as a consequence of increased replication forks during unperturbed cell growth was investigated. Transient pausing of the replisomes during replication stress impedes replication fork progression shortening track lengths of replicating fibers [19, 28] and inter-origin distances [29]. Indeed, measurement of track lengths of replicating DNA fibers [14, 30, 31] from isogenic mock-depleted (shGFP) or Onc-p53-depleted (shp53) H1975 (p53-R273H) human lung cancer cells (Fig. 1A) revealed shorter track lengths (Fig. 1B, C) and shorter inter-origin distances between contiguous origins (Fig. 1D, E) in shGFP H1975 cells in comparison to shp53 H1975 cells.

During replication stress, continued DNA unwinding and polymerase stalling generates single stranded (ssDNA) gaps, which allows chromatin loading of ssDNA-binding replication protein A (RPA) detected by RPA foci formation [22, 32]. Scoring of RPA foci revealed that in comparison to shGFP H1975 lung cancer cells, shp53 H1975 cells generate fewer RPA foci per nuclei and fewer nuclei with RPA foci over basal levels (Fig. 1F-I).

Stalling of active replisomes at the unwound replication fork during replication stress allows re-copying of once replicated DNA [19, 33]. Indeed, fiber analysis of replicating DNA in shGFP or shp53 H1975 cells after sequential labeling with IdU (red fluorescence) and CldU (green fluorescence) showed elongating CldU-labeled green DNA fibers that are copying once replicated IdU-labeled red DNA fibers generating yellow fibers in the merged images (Fig. 1J). Scoring of yellow fibers revealed fewer percentage of re-copied yellow fibers in shp53 H1975 cells compared to shGFP H1975 cells, (Fig. 1K).

Consistent with the above findings, depletion of p53 in H1048 (p53-R273C) increased track lengths of replicating fibers, reduced RPA foci formation and the frequency of re-copied yellow fibers (Fig. S1A–F). In contrast, stable expression of Onc-p53 mutants p53-R175H, p53-R273H or p53-R281G in H1299 (p53-null) lung cancer cells shortened track length of replicating DNA fibers compared to that observed in H1299 cells transfected with empty vector or stably expressing a deletion mutant (p53 del100-300), which lacks the mutated p53 allele (Fig. S1G, H). H1299 cells expressing p53-R175H, p53-R273H or p53-R281G also showed increased number of RPA foci per nuclei and frequency of re-copied replication forks compared to empty vector-transfected H1299 cells (Figs S1J–K). These results indicate that during DNA replication, Onc-p53 mutants impede replication fork progression, reduce inter-origin distances, promote chromatin loading of RPA and re-copying of once replicated DNA in a single S phase.

### Onc-p53 mutants increase co-localized 53BP1/H2AX foci formation in G1 nuclei and activate ATM signaling

**Fig. 2:**
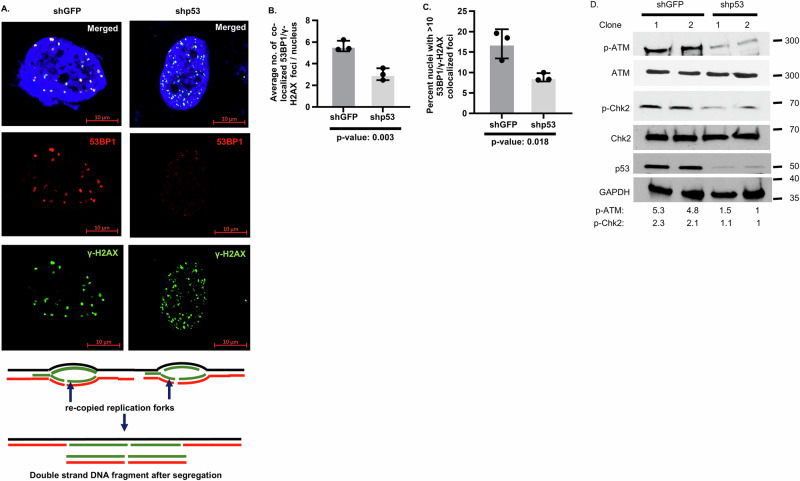
Onc-p53 mutants increase co-localized 53BP1/H2AX foci formation in G1 nuclei and activate ATM signaling. **A** Representative nuclei from shGFP or shp53 H1975 cells with co-localized 53BP1 (red) and γH2AX (green) foci is shown (Magnification ×40) in merged, red and green channels. Schematic explaining double strand DNA fragment generation from re-copied DNA sections are shown at the bottom panel. Bar graphs compare. **B** Average number of co-localized 53BP1 and γH2AX foci per cell, and **C** percent of nuclei with more than 10 co-localized 53BP1 and γH2AX foci in shGFP or shp53 H1975 cells. *p* values are shown. **D** Phospho (Ser1981)-ATM (p-ATM) and phospho (Thr68)-Chk2 (p-Chk2) levels in shGFP or shp53 H1975 cells detected by immunoblot analysis. Comparative levels determined by densitometry and normalized with loading control (GAPDH) are indicated at the bottom.

Mitotic transmission of re-copied replication forks generates DNA fragments that are transmitted to G1 nuclei after cell division and form co-localized 53BP1/γH2AX foci, whereas DNA strand breaks generated due to collapse of replication forks in S phase form γH2AX foci [14, 19, 34–38]. Scoring co-localized 53BP1/γH2AX foci, identified by lack of Cyclin A expression (Fig. S2A), revealed a robust reduction of 53BP1/γH2AX foci per nucleus and nuclei with 53BP1/γH2AX foci over basal levels in shp53 H1975 or H1048 cells, compared to respective shGFP cells (Fig. 2A–C, S2B–D). In agreement with this observation, p53-null H1299 cells stably expressing p53-R175H or p53-R273H showed increased 53BP1/γH2AX foci compared to vector-transfected H1299 cells (Fig. S2E, F). These data indicate that Onc-p53 increases 53BP1/γH2AX foci formation, indicating presence of DNA fragments. Consistent with our published report [14] p53-depleted cells (shp53), which are more vulnerable to replication fork collapse compared to mock-depleted (shGFP) cells, primarily formed γH2AX foci related to fork collapse in the S phase (Fig. 2A, S2B).

We determined whether DNA fragments generated from re-copied replication forks after mitotic transmission activate ATM phosphorylation at Ser1981 [39]. Indeed, extracts from unchallenged asynchronously growing shp53 H1975 and H1048 cells showed a robust decline in phospho (Ser1981)-ATM (p-ATM) and its target phospho (Thr68) Chk2 (p-Chk2) [40] levels compared to that from shGFP H1975 and H1048 cells (Fig. 2D, S2G), without changing total ATM or Chk2 levels significantly. Conversely, stable expressions of p53-R273H or p53-R175H mutants in p53-null H1299 cells showed elevated levels of p-ATM compared to H1299 cells with empty vector, whereas Onc-p53 del 100-300, lacking mutated Onc-p53 allele did not phosphorylate ATM (Fig. S2H). Thus, Onc-p53 induces ATM signaling during normal unchallenged cell growth.

### Release of replication stress driven by Onc-p53 reduces the frequency of re-copied replication forks, 53BP1/γH2AX foci formation in G1 nuclei and ATM phosphorylation

**Fig. 3:**
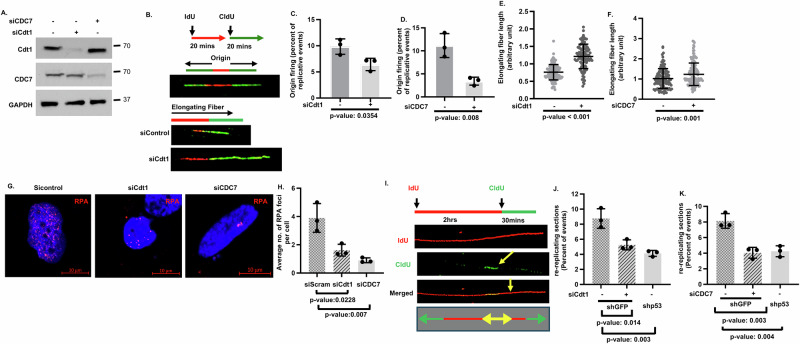
Reduction in frequency of origin firing rescues retardation of fork progression, reduces chromatin loading of RPA and frequency of re-copied replication forks. Immunoblot analysis (**A**) to ensure partial depletion of Cdt1 using siCdt1, and CDC7 using siCDC7 in H1975 cells. Control siRNA was used for mock-depletion (shown as –). GAPDH is a loading control. **B** Representative confocal images (Magnification ×63) of bi-directional origins and replicating DNA fibers undergoing elongation from siControl (–) or siCdt1-transfeced cells in early S phase. Bar graphs compare the frequency of origin firing normalized by total replicative events in (**C**) siCdt1 or (**D**) siCDC7-transfected cells compared to sicontrol (–) transfected cells. Swarm plots compare elongating fiber length in (**E**) siCdt1 or (**F**) siCDC7-transfected cells (+) with sicontrol-transfected cells (–). **G** Representative confocal images (Magnification ×40) of nuclei with RPA foci. **H** Bar graph comparing average number of RPA foci per nuclei in sicontrol, siCdt1 and siCDC7-transfected H1975 cells. **I** Confocal image of a representative re-copied replication fork (indicated by arrows) in replicating DNA fibers sequentially pulse labeled with IdU (red) and CldU (green) as described (upper panel **I**) shown in red, green, and merged channels The bar graphs compare percent of fibers re-copying once replicated DNA strand in (**J**) siCdt1- or (**K**) siCDC7- (+) or sicontrol-transfected shGFP H1975 (–) or shp53 H1975 (–) cells. *p* values are shown.

To determine whether re-copied replication forks driven by Onc-p53 promotes 53BP1/γH2AX foci formation in G1 nuclei and ATM phosphorylation, the frequency of replication origin firing was reduced in H1975 cells using two different, previously demonstrated [41, 42] siRNAs that partially depletes two different replication factors, the licensing factor Cdt1 [43] and DBF4-dependent kinase CDC7 that knock down origin firing. The results revealed that guaranteed on-target siCdt1 or siCDC7 siRNA pools, reduced Cdt1 or CDC7 expression (Fig. 3A) and consequently knocked down origin firing as expected (Fig. 3B-D). Reduced frequency of origin firing released replication stress as evidenced by increased track lengths of elongating fibers (Fig. 3E, F), reduced RPA foci formation (Fig. 3G, H) and frequency of re-copied replication forks (Fig. 3I–K) compared to control siRNA-transfected cells despite the presence of p53-R273H in H1975 cells.

**Fig. 4:**
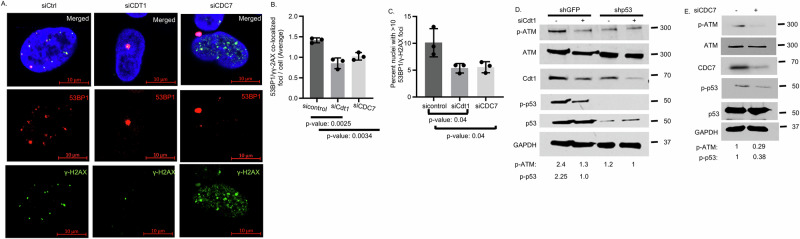
Reduced frequency of re-copied replication forks reduces co-localized 53BP1/H2AX foci formation and ATM phosphorylation. **A** Representative nuclei from mock-depleted (siCtrl), Cdt1-depleted (siCdt1) or CDC7-depleted (siCDC7) H1975 cells with co-localized 53BP1(red) and γH2AX (green) foci is shown (Magnification 40x) in merged, red and green channels. Bar graphs compare (**B**) average number of co-localized 53BP1 and γH2AX foci per cell, and **C** percent of nuclei with more than 10 co-localized 53BP1 and γH2AX foci in H1975 cells transfected with sicontrol, siCdt1 or siCDC7 RNA. *p* values are shown. Immunoblot analysis to detect phospho (Ser1981)-ATM (p-ATM) and phospho (Ser15)-p53 (p-p53) levels in shGFP or shp53 H1975 cells transfected with (**D**) sicontrol (–) or siCdt1, and (**E**) sicontrol (–) or siCDC7 (+) RNA. Comparative levels determined by densitometry and normalized by loading control GAPDH are indicated at the bottom.

Reduced origin firing by the siRNAs also reduced co-localized 53BP1/γH2AX foci formation in H1975 cells (Fig. 4A–C). Since siCDC7 directly inhibits origin firing, it induced γH2AX foci formation due to fork collapse and DNA fragmentation in S phase (Fig. 4A). The knockdown of origin firing by the siRNAs reduced p-ATM and p (Ser15)-p53 levels in shGFP H1975 cell extracts (Fig. 4D, E) in comparison to control siRNA-transfected cells. As expected, siCdt1 did not alter p-ATM levels in shp53 H1975 cells. Additionally, siCdt1 in H1299 cells stably expressing p53-R273H caused downregulation of p-ATM and p-p53 in comparison to control siRNA-transfected cells, whereas p-ATM levels remain unaltered in siCdt1-transfected H1299 cells stably transfected with empty vector (Fig. S3). These data indicate that Onc-p53-driven replication stress is necessary and sufficient to induce ATM signaling and Onc-p53 phosphorylation in lung cancer cells expressing Onc-p53 endogenously or stably.

### Lung cancer cells with p53 mutation show Onc-p53 dependent genome segregation errors

**Fig. 5:**
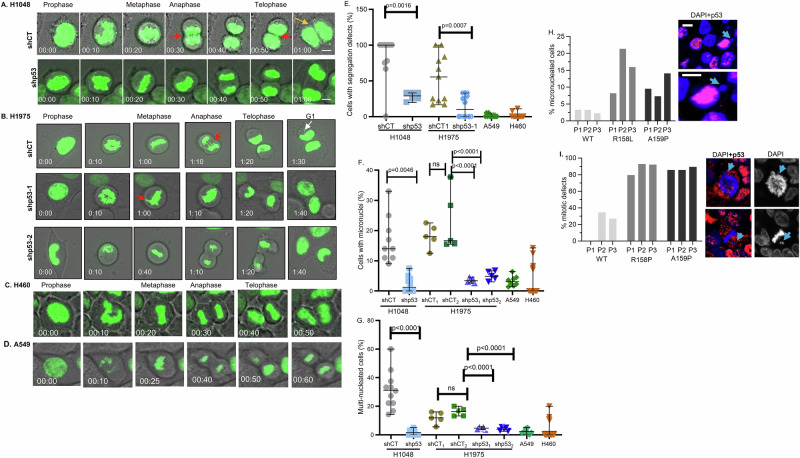
Onc-p53 induces and selects for mitotic segregation errors in lung cancer cells and patient derived lung tumors. Time-lapse live images and videos (supplementary data attached) of mock depleted (shCT) and p53-depleted (shp53) (**A**) H1048, **B** mock depleted (shCT) and p53-depleted (shp53) H1975, **C** H460, **D** A549 lung cancer cell lines stably expressing GFP-tagged H2B, monitored (5 min/image) as cells enter and exit mitosis. Chromatin bridge and lagging chromosomes (red arrows), binucleated cells (yellow arrows); and micronuclei (white arrow) are indicated. Scale, 10 µm. Scatter plots compare percentage of cell with segregation defects (**E**), micronuclei (**F**) and multi nuclei (**G**) in indicated cell lines. Fifty cells/condition were used, repeated twice. *p* values are shown. **H**: FFPE sections from primary (P1) and serially passaged (P2, P3) patient-derived NSCLC tumors harboring WT p53 or p53 mutants R158L and R159P were immunostained with anti-p53 antibody and DAPI. Micronuclei and mitotic defects in cells with high levels of p53 were analyzed. Bar graphs show percentages of tumor cells with (**H**) micronuclei and (**I**) mitotic cells with chromosomal abnormalities in WT p53 and R158L and R159P expressing tumors. Right panels show immunostaining (Alexa 594) of tumor samples harboring R158L and R159P mutants and DAPI staining (blue) of nuclei, associated micronuclei, and chromosomal abnormalities (yellow arrows). For **H** 200–300 cells, and for **I** 14–52 mitotic cells were counted (Table S1). The presence of mitotic cells in P1 tumor sections with WT p53 were below the level of detection. Scale: 10 µm.

The presence of DNA fragments generated from re-copied DNA replication forks (Fig. 2) may lead to their aberrant alignment and processing during anaphase segregation generating micronuclei, lagging chromosomes, and eventually bi- or multi-nucleated cells as a result of cytokinetic delay [25–27]. The contribution of Onc-p53 to create genome segregation errors was investigated by time lapse live video imaging of lung cancer cells stably expressing GFP-tagged histone H2B to allow fluorescent tracking of cell division. Scoring of cells displaying lagging chromosomes, micronuclei, and multiple nuclei (Fig. 5A–G, S4A, videos) revealed that shCT H1048 and H1975 cells, which show a higher frequency of re-copied replication forks than isogenic shp53 cells, (Fig. 1, S1F) exhibit robust increase in percentages of cells with segregation errors and cytokinetic defects compared to isogenic shp53 cells or A549 and H460 cells with WT p53 (Fig. 5E–G, S4A). These observations indicate that, in addition to genome instability reported in the absence of WT p53 function [44, 45], Onc-p53 triggers mitotic segregation errors and formation of multi-nuclear cells not displayed by p53-depleted cells, strongly implicating Onc-p53 induced re-copied replication intermediates in the aberrant cell division despite generating segregation errors.

### In contrast to patient-derived lung tumors with WT p53, lung tumors with Onc-p53 show selection of cells with mitotic aberration

Since Onc-p53 induced mitotic segregation errors (Fig. 5), frequency of mitotic aberration in propagating tumor cells with stabilized Onc-p53 was determined. De-identified human lung tumor xenografts (PDX) with Onc-p53 mutated in the DNA-binding domain R158L or A159P [46, 47] or WT p53 were propagated by serial passaging. FFPE sections from original tumors (P1) and serially passaged (P2, P3) PDXs were analyzed. As expected [48, 49], R158L and A159P tumors showed higher levels p53 expression than tumors with WT p53 (Fig. 5H, I). Mitotic aberration in tumor cells with stabilized p53 (R158L or A159P) from original (P1) or passaged (P2, P3) lung tumors were counted. Higher percentage of cells with micronuclei (Fig. 5H) was observed in P2 and P3 tumors compared to P1 tumors with Onc-p53, whereas almost all cells in P1 to P3 tumors showed mitotic aberration (Fig. 5I, S4). Consistent with published reports, cells from original or passaged lung tumor samples (P1, P2 P3) with WT p53 had fewer mitotic aberration, which did not select for mitotic aberration in subsequent passages, arguing for a selective advantage for cells with segregation errors. (Fig. 5I, Table S1). These data demonstrate that in contrast to lung tumors with WT p53, lung tumors with Onc-p53 selects for tumor cells with chromosome segregation errors during serial passages signifying their selective advantage during tumor growth.

### Onc-p53 induces ATM signaling and stabilizes itself, in turn upregulating Cyclin A and Chk1 expression establishing a self-sustaining feedforward loop

**Fig. 6:**
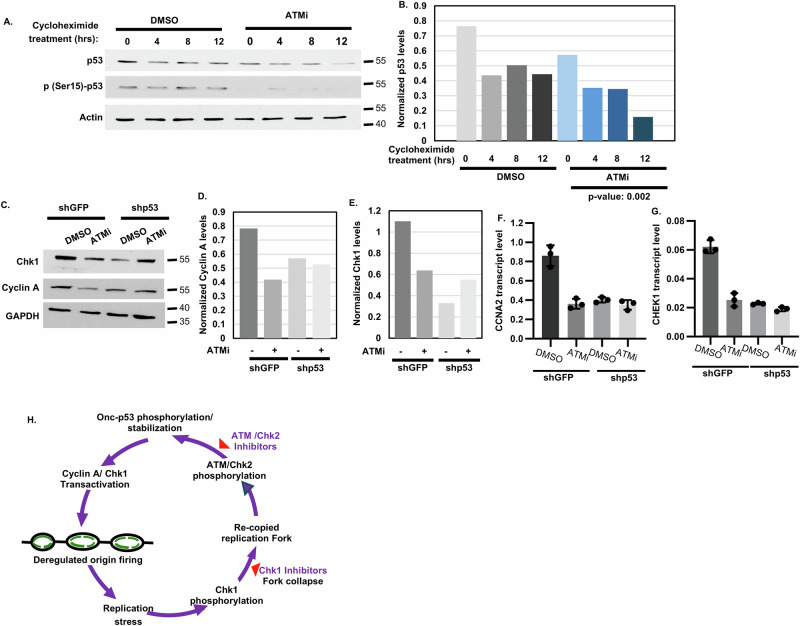
Onc-p53 activates ATM signaling stabilizing itself, in-turn upregulating Cyclin A and Chk1 expression. **A** Immunoblot analysis to detect p53 levels in extracts of H1975 cells after cycloheximide chase at the indicated time points in the presence of vehicle (DMSO) or an ATM inhibitor, KU-600019, (ATMi). Actin was used as a loading control. **B** Bar graphs show densitometric analysis of total p53 levels normalized with loading control obtained from the immunoblot in (**A**). *p* value is shown at the bottom. **C** Immunoblot analysis showing Chk1 and Cyclin A expression in extracts from shGFP and shp53 H1975 cells at the early S phase in the presence of DMSO or ATMi. GAPDH was used as a loading control. Densitometric analysis of (**D**) Cyclin A and (**E**) Chk1 levels normalized with loading control obtained from the immunoblot in (**C**). Expression of (**F**) CCNA2 and (**G**) CHEK1 transcript levels normalized with GAPDH in shGFP and shp53 H1975 cells at the early S phase in the presence of DMSO or ATMi. The bar graphs (**F**,** G**) are plotted as mean ± SD of three independent experiments. **H** Proposed mechanisms of p53 accumulation and targeting of tumor growth.

Since in cancer cells Onc-p53 mutants have a long half-life, which allows their accumulation [50, 51], the contribution of Onc-p53-induced ATM signaling (Fig. 2D) to the half-life of Onc-p53 was investigated using cycloheximide chase assay [52]. In agreement with the ability of cycloheximide to inhibit protein synthesis [53], immunoblot analysis of H1975 cells treated with cycloheximide showed a moderate reduction in p53 levels within 4 h in both vehicle (DMSO)- or ATM inhibitor, KU60091 (ATMi)-treated cells. However, unlike DMSO treated cells, which did not show further decline in p53 levels, the ATMi-treated cells induced progressive decline (73% from 4 to 12 h) in p53 levels upon cycloheximide exposure (Fig. 6A, B). Demonstrating on-target activity, ATMi reduced p-(Ser15)-p53 levels (Fig. 6A), confirming that in H1975 cells ATM kinase phosphorylates and stabilizes Onc-p53.

Validating Onc-p53 stabilization, shGFP H1975 cells expressed higher levels of Cyclin A and Chk1 protein (Fig. 6C–E) and RNA (Fig. 6F, G) compared to shp53 H1975 cells. ATMi treatment caused a robust decline in Cyclin A and Chk1 expression in shGFP H1975 cells but not in shp53 H1975 cells. Supporting this finding, ATMi also reduced CCNA2 and CHEK1 gene expressions in p53-null H1299 cells stably expressing Onc-p53 R273H compared to empty vector-transfected H1299 cells (Fig. S5A, B). These data indicate that ATM inhibition diminishes Onc-p53-induced Cyclin A and Chk1 expression.

These data strongly argue that upregulation of Cyclin A and Chk1 expression by Onc-p53 (Fig. 6C–G, S5) increases frequency of replication origin firing [14], generates re-copied replication forks (Fig. 1, S1), inducing ATM kinase activity (Fig. 2D S2G, H), and in-turn phosphorylation and stabilization of Onc-p53 (Fig. 6A,B) thus, generating a feed forward loop (Fig. 6H) leading to a continuous regeneration of functionally active Onc-p53.

### Release of replication stress induces apoptosis specifically in lung cancer cells with Onc-p53

Onc-p53 induces Chk1 expression that prevent collapse of unscheduled replication forks it generates [14], thus causing replication stress and induction of ATM signaling (Figs. 1–4). Since inhibition of Chk1 activity induces replication fork collapse [14, 54], Chk1 and ATM kinase inhibitors were used as tools to investigate dependency of lung cancer cells with Onc p53 on replication stress and induction of ATM signaling.

**Fig. 7:**
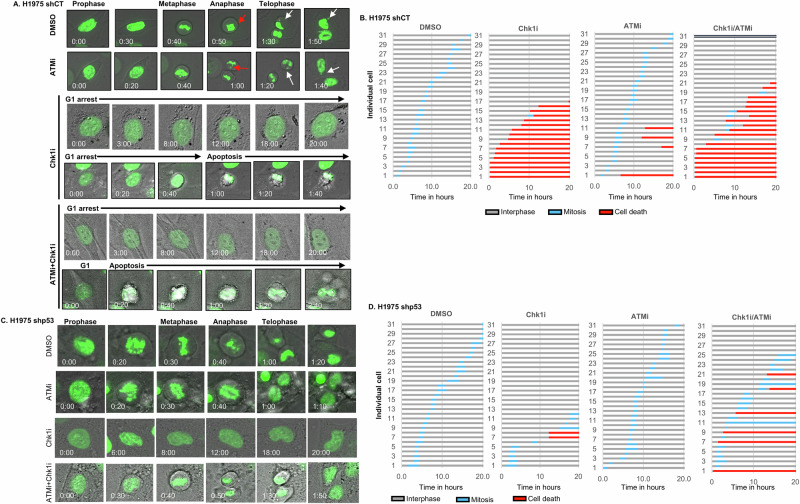
Release of replication stress using Chk1 inhibitor induces apoptosis specifically in lung cancer cells with Onc-p53. Isogenic shCT or shp53 H1975 cells were treated with vehicle (DMSO), or Chk1i (MK8776 10uM) and/or ATMi (KU-600019 1uM) for 24 h. Cell proliferation was then monitored by video microscopy (5 min/image). Representative time-lapse images and videos (bottom) of H1975 (**A**) shCT and (**B**) shp53 cells stably expressing GFP-tagged H2B treated with DMSO, ATMi and/or Chk1i alone or in combination. Micronuclei and chromosome segregation defects are indicated by white and red arrows respectively. Scale of the images is 10 µm. **C**,** D** Horizontal bar graphs show time spent by individual cells in interphase (gray), mitosis (blue) before entering cell death (red) during cell cycle progression. Thirty individual cells were monitored after indicated treatments.

Live imaging of H2B-GFP expressing isogenic mock-depleted (H1975 shCT) and p53-depleted H1975 cells (H1975 shp53) cells treated with vehicle (DMSO) or Chk1 inhibitor MK8776 [55] (Chk1i) and/or ATM inhibitor, KU-60019, (ATMi) revealed that Chk1i-treated H1975 shCT cells were either arrested (approximately 45% of in interphase), or underwent apoptosis (approximately 55%) before mitotic entry (Fig. 7A,B 7A video). In contrast, Chk1i-treated H1975 shp53 cells stalled in interphase and displayed delayed mitotic entry as expected in cells with fewer active replication forks in the presence of Chk1i but did not show significant cell death (Fig. 7C, D, video). Thus, Onc-p53 introduces vulnerability to Chk1 inhibition in lung cancer cells, requiring its ability to induce Chk1 expression for their survival and proliferation.

Furthermore, we further challenged replication stress with an inhibitor of Aurora kinase B (AURKB), Barasertib. Onc-p53 upregulates AURKB [16], which is required for cytokinesis in cells with elevated replication stress [56]. Accordingly, an AURKB inhibitor, Barasertib, preferentially blocked cytokinesis, and proliferation of mock-depleted shGFP H1975 cells over p53-depleted shp53 H1975 cells (Fig S6A–C). Thus, Chk1 or AURKB inhibitors block the pathway before transmission of the re-copied replication intermediate to G1 nuclei required for inducing ATM signaling. However, ATMi alone or in combination with Chk1i showed a minor (13–20%) increase in H1975 shCT cell death, whereas H1975 shp53 cells did not show significant stalling or apoptotic cell death in the presence of ATMi, (Fig. 7B, D) indicating that Onc-p53-induced ATM activation does not have a major contribution during mitotic entry of cultured cells.

### Small molecule inhibitors of ATM or its downstream target Chk2 cooperate with Chk1 inhibitors to specifically inhibit growth of cultured lung cancer cells or xenograft tumors with Onc-p53 inducing apoptosis

**Fig. 8:**
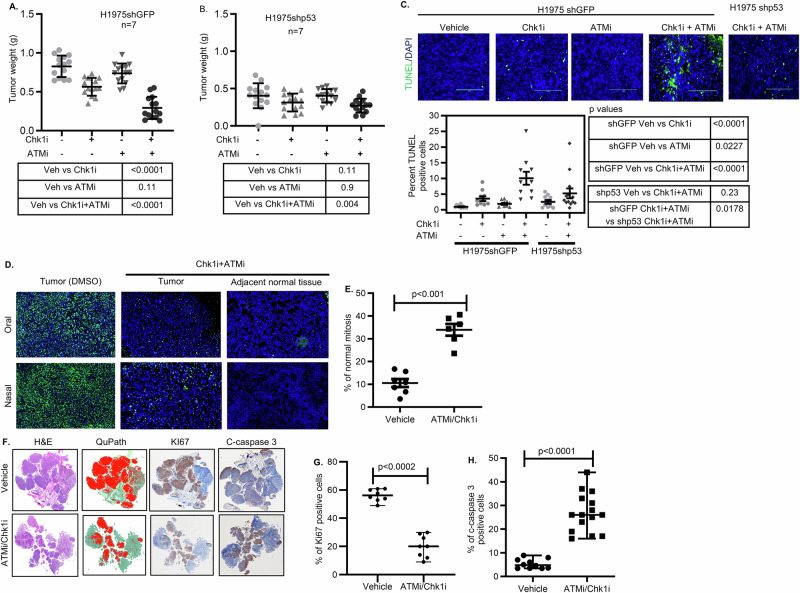
Small molecule inhibitors of Chk1 and ATM cooperate with each other to preferentially inhibit proliferation of H1975 xenograft tumors with mutant p53. Scatter plots of tumor weights from xenografts of H1975 (**A**) shGFP and **B** shp53 cells after indicated vehicle or drug treatments. Seven mice were used in each group. *p* values (two-tailed *t* test) are shown. TUNEL assay for apoptosis (**C**) in tumors from (**A** and **B**). Representative images (Magnification ×20) and Swarm plot (lower panel) of vehicle (–), Chk1i and/or ATMi -treated tumor sections from H1975 shGFP and shp53 xenografts show apoptotic cells (green fluorescence) by TUNEL assay. At least 10 blind images were used. Representative data from three different mice tumor sections were counted. *p* values are shown. **D** Representative image of anti-p53 immunostaining and DAPI staining of FFPE lung tumor or lung tissue sections after vehicle- or ATMi/Chk1i-treatment (oral or nasal) from orthotopic H1975 xenograft tumors expressing luciferase (Fig. S7). **E** Scatter plots show the percentage of normal mitotic cells in (**D**). **F** Whole slide images of lung tumors after H&E staining, Ki67 or c-Caspase 3 immunostaining, and QuPath image (Green: stroma, dark red, tumor, blue normal lung tissue). Scatter plots (bottom panel) show the percentage of (**G**) Ki67 and **H** c-caspase 3 positive cells in (**F**) from tumors of two mice. *p* values are shown.

Since activation of ATM kinase by Onc-p53 increases the stability of Onc-p53 (Fig. 6), we reasoned that depletion of the high levels of accumulated Onc-p53 in lung cancer cells by destabilization may require ATMi treatment spanning several rounds of cell proliferation. Indeed, proliferation of isogenic shGFP or shp53 H1975 cells treated with vehicle (DMSO), or Chk1i and/or ATMi over a period of 7 days showed synergistic growth inhibition [57] of shGFP H1975 cells with a combination Index (CI) of 0.2. In contrast, shp53 H1975 cells showed a minor decline in growth rate after Chk1i or/and ATMi treatment (Fig. S6D–F). WI38 normal embryonic lung fibroblast cells that harbor WT p53 also showed a limited decline in growth rate by either Chk1i or ATMi treatment (Fig.S6G, H) with no additional effects of combining the inhibitors. Furthermore, combined Chk1i and ATMi generated a robust 73% inhibition of H1975 shGFP subcutaneous xenograft tumor growth at a dose when Chk1i or ATMi alone showed 36% and 12% inhibition respectively (Fig. 8A,). Growth of H1975 shp53 cells or xenograft tumors showed a muted response to individual or combined inhibitor treatment (Fig. 8B), and Xenograft tumors from H460 lung cancer cells, which harbor WT p53, proliferated abundantly in the presence or absence of Chk1i and/or ATMi (Fig. S7A). Replacing ATMi, inhibitor of Chk2, a phosphorylation target of ATM, showed similar synergistic inhibition of shGFP H1975 cell (Combination Index: 0.5) and tumor growth of cells (Fig. S8A–E).

Supporting our data from live imaging (Fig. 7), FFPE tumor sections from shGFP H1975 xenograft tumors treated with combined Chk1i and ATMi or Chk2i revealed robust increases in TUNEL stained cells after treatment relative to vehicle treated tumors (Fig. 8C, S8F, G) indicating apoptotic cell death. Chk1i treated tumor sections showed a small but statistically significant increase in TUNEL stained cells compared to vehicle treated tumor sections, whereas ATMi or Chk2i alone did not show this difference. Chk1i and ATMi or Chk2i induced only a nominal increase in apoptosis in H1975 shp53 tumors. These data indicate that tumor growth driven by Onc-p53 could be eliminated efficiently and selectively releasing replication stress by combined application of Chk1 and ATM/Chk2 inhibitors.

The treatment of mice bearing pre-formed lung tumors generated by orthotopic implantation of H1975 cells expressing luciferase (H1975-Luc) with combined Chk1i and ATMi either by nasal or oral route caused a drastic decrease in total bioluminescent flux from both lung primary tumors and liver metastasis (Fig. S7B, C). FFPE tumor sections showed near-complete removal of cells with high Onc-p53 levels (Fig. 8D). Scoring of mitotic cells obtained from DAPI stained nuclei in tumor sections showed increased frequency of cells with normal mitosis in Onc-p53-expressing cells (Fig. 8E). The whole slide image analysis showed a decrease in Ki67 and an increase in cleaved-caspase-3 (c-caspase-3) immunostained cells after treatment with Chk1i and ATMi (Fig. 8F–H). These data indicate that releasing replication stress using combined Chk1 and ATM inhibitors eliminate Onc-p53 expressing tumor cells with mitotic aberration.

## Discussion

Current literature reports that p53 mutants frequently gain oncogenic functions (Onc-p53), where the tumors or cancer cells require the presence of the mutated p53 allele for survival and proliferation [12, 14, 18, 58, 59]. A recent report has suggested that removal of Onc-p53 allele does not alter its oncogenic property [60]. Our recent data (Fig S9) revealed that in short-term (approximately 3 weeks) tumor formation experiments, H1975 p53-Crispr control and Crispr knockout (KO) of p53-R273H in H1975 human lung cancer cells show marginal difference. However, allowing longer time for tumor growth (approximately 6 weeks) H1975 p53-Crispr KO of lung cancer cells abolishes its tumor formation ability revealing that tumor formation by H1975 p53-Crispr control cells require longer time (approximately 6 weeks) for growth whereas tumors from H1975 p53-Crispr KO cells do not grow any further from 3 weeks.

Tumors or cancer cells with Onc-p53 mutation are often associated with chromosomal abnormalities as determined by copy number changes or aneuploidy [44, 45]. Additionally, recent studies [61, 62] report a correlation between mutant p53 and chromosome break. The rationale for increased frequency of chromosomal abnormalities in cancer cells with on Onc-p53 during tumor survival and progression remains unclear.

We have reported earlier that Onc-p53 increases frequency of DNA replication origin firing [14]. This article demonstrate that increases in the frequency of replication origin firing by Onc-p53 during unperturbed proliferation of lung or lung cancer cells [14] develops replication stress generating re-copied replication intermediates inducing ATM and p53 phosphorylation and thus Onc-p53 stabilization, which can be specifically targeted using Chk1/ATM inhibitors. Development of replication stress [19] is evidenced by retarded replication fork progression, reduced inter-origin distances, increased RPA loading and increased frequency of re-copied replication forks (Fig. 1). While replication fork asymmetry has been implicated in replication stress [63], we did not use the criterion because frequency of replication fork collapse is higher in p53-null cells compared to cell with Onc-p53 [14]. The mutated allele of Onc-p53 required for its tumor formation ability [18], was also required for development of replication stress (Fig. S1H) implicating Onc-p53-induced replication stress in tumor formation.

This investigation revealed that the replication intermediates generated from re-copied replication forks induced by Onc-p53 are transmitted to G1 nuclei of daughter cells, causing formation of 53BP1 foci mostly co-localized with γ-H2AX foci, and activate ATM signaling (Figs. 2–4) during unperturbed proliferation of cancer cells in the absence of any exogenous DNA damaging agent. This mechanism of ATM signaling is different from radiation induced short-term ATM signaling. It has been reported that p53 mutants interfere with MRN complex formation and ATM signaling activated by radiation induced indiscriminate DNA breakage [64]. DNA fragments generated from re-copied replication forks during unchallenged proliferation of cells with p53 mutation activated ATM signaling efficiently in the absence of any genotoxic treatment, suggesting different mechanism involved in replication stress-induced and radiation-induced ATM activation [39]. Our data show that generation of re-copied fragments in each replication cycle, consequent ATM activation, Onc-p53 phosphorylation and stabilization emboldens oncogenic function of p53, which in-turn increases origin firing, creating a feed forward cycle (Fig. 6H). Thus, ATM activation becomes an important node that reinforces tumor formation by Onc-p53. We, thus, demonstrate a novel mechanism of Onc-p53 stabilization in cancer cells in addition to the current concepts that Onc-p53 owes its long half-life to its interaction with heat shock proteins that protect Onc-p53 from E3 ubiquitin ligases [12, 49, 65]. Since Onc-p53 does not upregulate ATR expression, cancer cells with Onc-p53 do not show dependency on ATR. ATR inhibitors induce cell death irrespective of p53 status (data not shown).

In agreement with reports that unresolved DNA fragments interfere with chromosome alignment and processing during anaphase segregation [27], our time lapse video microscopy revealed that DNA fragments generated from Onc-p53 induced re-copied replication forks create and select for cells with mitotic segregation errors during tumor development (Fig. 5A-I, TableS1, videos).

Selection of lung cancer cells with chromosomal aberration by Onc-p53 thus seems to be due to stabilization of Onc-p53 by re-copied replication forks, which constitutes an Achilles heel during oncogenesis. Our live imaging experiments revealed in real time that release of replication stress with a Chk1 inhibitor, which induces replication fork collapse [14], specifically triggers apoptotic death of Onc-p53 expressing cells, while the inhibitors slightly delay mitotic entry of p53-depleted cells emphasizing the dependency of the lung cancer cells harboring Onc-p53 on replication stress (Fig. 7A-D, Videos). Combined application of ATM and Chk1 inhibitors prompted not only robust apoptotic cell death in orthotopic xenograft tumors with Onc-p53, but also a near-complete elimination of cancer cells expressing Onc-p53 in lung orthotopic tumors (Fig. 8). Thus, breaking the self-reinforcing feedforward loop (Fig. 6H) that drives replication stress-induced mitotic aberrations and heightened Onc-p53 stability eliminates cells that acquired mitotic aberration and selective growth advantage. Since absence of Onc-p53 in lung or lung cancer cells do not cause replication stress, they display a slow tumor growth unaffected by Chk1 or ATM inhibitors. While not studied in the context of Onc-p53, toxicity of Chk1 and ATM inhibitors have been tested as monotherapy in clinical trials. Cooperativity between Chk1 and ATM kinase inhibitors suggest increased efficacy of combined therapy, which may allow one to use lower dosage thus reducing toxicity. These findings provide compelling evidence that targeting the oncogenic properties of mutant p53 has tumor specific therapeutic potential of cell cycle checkpoint inhibitors.

## Materials and methods

### Plasmids, lentiviral vectors, and cell lines

Generation of plasmids, lentiviruses and stable transfectants expressing Onc-p53 or shRNA against GFP or p53 has been carried out using pLKO.1 expression vector purchased from Open Biosystem (Lafayette, Co) following supplier’s protocols. Expression vector pEGFP-N1, purchase from Addgene, was used for expression of GFP-tagged H2B (H2B-GFP). H1299, H1975 and H1048 cell lines were obtained from American Type Culture Collection (ATCC) as published earlier. H1975 p53-Crispr control and Crispr knockout (KO) cells are generous gifts of Hisashi Harada [66].

### Chemicals and drugs

Iododeoxyuridine (IdU) and chlorodeoxyuridine (CldU) were purchased from Sigma. PHA767491 (Tocris Bioscience) was used at a concentration of 1.5 μM for indicated times [67]. ATM inhibitor KU60019 (Selleck Chem) was used at a concentration of 3 µM for treatment of cultured cells for indicated times [68]. Chk1 inhibitor MK8776 (Selleck Chem) was used at a concentration of 5-20 µM for treatment of cultured cells for indicated times [69]. Chk2 inhibitor II hydrate (Sigma) was used at a concentration of 5 µM for treatment of cultured cells [70]. ATM inhibitor AZD0156 (Selleck Chem) was used at a concentration of 1 µM for treatment of cultured cells for indicated times. The CDC7 inhibitory activity of PHA767491 was confirmed by ensuring a decrease in MCM2 phosphorylation at Ser40/41 by immunoblot analysis [67] (Fig. S4A).

### Antibodies

Antibodies used included anti-p53 (sc-123), Chk1 (sc-56288), Cyclin A (sc-751), GAPDH (sc-47724), γ Tubulin Antibody (C-11), from Santa-Cruz Biotechnology, phospho Chk2 Thr68 (2197), phosphor-p53 ser15 (82530), Chk2 (6334), γ-H2AX (9718) and 53BP1 (88439) from Cell Signaling Technology, used according to manufacturer’s protocol. For DNA fiber analysis, IdU was detected by mouse anti-bromodeoxyuridine (347580) from Becton Dickinson primary antibody and Alexafluor 594-conjugated rabbit anti-mouse and Alexafluor 594-conjugated goat anti-rabbit (Life Technologies) secondary and tertiary antibodies, respectively. CldU was detected by rat anti-bromodeoxyuridine (OBT0030G) primary antibody from Accurate, and Alexafluor 488-conjugated chicken anti-rat and Alexafluor 488-conjugated goat anti-chicken (Life Technologies) secondary antibodies. Slides were mounted using ProLong Gold anti-fade reagent (Molecular Probes).

### Cell synchronization

Cells were partially synchronized by density arrest followed by replating. For identifying newly initiated DNA replication origins and elongating DNA fibers, cells were examined at 12 h after replating. For identifying re-replicating DNA fibers, cluster origins and RPA foci, cells were harvested at 16 h after replating. For 53bp1/γ-H2AX foci cells were analyzed at 24 h post replating.

### Detection of 53BP1/γ-H2AX, RPA foci

For detection of foci, cultured cells were plated on coverslips and treated with permeabilization buffer prior to fixing using 3% paraformaldehyde following published protocol [71]. The cells were then treated with 0.5% triton X-100 for 10 min, followed by PBS washes and 1 h blocking in 5% BSA. The primary antibody incubation was performed in the blocking buffer overnight at 4 °C followed by PBS wash and 1-h incubation with Alexa 488-conjugated secondary antibody. Coverslips were mounted on slides using Prolong Gold Antifade with DAPI and imaged by confocal microscopy (Zeiss LSM700) at ×40 magnification. 50–60 nuclei were randomly scored for foci using Foci Counter software. For identification of 53BP1 foci in G1 nuclei, cells were immunostained with anti-53BP1 (Alexa 594), anti-Cyclin A (Alexa 488). 53BP1 foci in cells not stained with cyclin A were counted.

### DNA fiber analysis

DNA replication origin firing was determined by DNA fiber spreading analysis following published protocols [14, 30, 31]. Briefly, cells were pulse-labeled sequentially with nucleotide analogs IdU (50 μM) and CIdU (100 μM) to track the replication pattern and directionality of fork movement. Cells were collected by trypsinization, and genomic DNA of approximately 600 cells was aligned on slides by fiber spreading as described earlier. The slides were then air dried and fixed 3:1 methanol/acetic acid and dried overnight. After acid treatment (2.5 N HCl 30 min) and blocking (2% BSA in PBS), DNA fibers on slides were immunostained with primary antibodies against IdU and CIdU followed by fluorescently labeled secondary and tertiary antibodies, washed dried and mounted in Prolong Gold Antifade (Life Technologies). Images were collected by confocal microscopy (Zeiss LSM700). Newly initiated single origins were detected as red tracks flanked on both sides by green tracks. Approximately 150–200 untangled fibers and 40 contiguous bidirectional origins from each sample were scored and analyzed using Image J software (NIH).

For identifying re-replicating fibers, IdU (50 μM) was incorporated for 2 h followed by CIdU (100 μM) for 30 min. Cells were harvested and processed as described above. Re-replicating origins were identified as green tracks superimposed on red tracks appearing as yellow fibers flanked by red tracks on both ends [19, 33]. Approximately 200 origins from each sample were scored and analyzed using Image J software (NIH).

### Live cell imaging

Imaging was performed using an inverted microscope (Zeiss Cell Observer Spinning Disc confocal microscope) controlled by Zeiss software and equipped with a Yokogawa CSU-X1A spinning disc unit, 2 Photometrics Evolve 512 cooled emCCD cameras, and laser illumination system. For chromosome segregation analysis, GFP-H2B-positive cells were seeded on glass-bottom dishes (μ-Dish, IBIDI), and cultured in DMEM media without phenol red, +10% FBS (Life Technologies). For lung cancer cells with Onc-p53, isogenic mock-depleted (shCT) and p53-depleted cells labeled with H2B-GFP were used. Treatment with vehicle (DMSO) or Chk1 inhibitor MK8776 [55] (Chk1i) and/or ATM inhibitor, KU-60019, (ATMi), are shown. Mitotic entry, defined by nuclear envelop breakdown, was monitored. Cells were recorded using a 40×/NA 1.30 oil immersion lens at 1 image every 3 min for 24 h. For proliferation imaging assay, cells were imaged with a 10×/NA 0.3 phase 1 lens for 24 h at one image every 5 min. Analysis and quantification were performed with ImageJ software (NIH).

### RNA extraction *and qPCR*

RNA extraction, cDNA preparation and QPCR were performed following standard protocols using the Thermoscript Reverse Transcription-PCR system (Life Technologies). QPCR was carried out using a LightCycler system (Roche). Primers[14] were designed using OLIGO 5 software (Molecular Biology Insights) and were synthesized by Integrated DNA Technologies. Human GAPDH fwd - 5ʹ-GTCAACGGATTTGGT CGTATT-3ʹ, rev - 5ʹ- GATCTCGCTCCTGGAAGATGG-3ʹ, human CHEK1 fwd - 5ʹ-GAAAGG GGCAAAAAGG-3ʹ, rev - 5ʹ-ATGTATGAGGGGCTGGTA-3ʹ, human CCNA2 fwd - 5ʹ-GACGGC GCTCCAAGAGG-3ʹ, rev - 5ʹ-AATGGTGAACGCAGGCTGTT-3ʹ. Reactions were performed in triplicate utilizing SYBR green dye, which exhibits a higher fluorescence upon binding of double-stranded DNA. The methods have been described previously [14, 72]. Reactions were performed in duplicate and repeated in three independent sets of experiments.

### Determination of mutant p53 stability

Stability of endogenous Onc-p53 in H1975 lung cancer cells in the presence or absence of ATM inhibitor was determined by cycloheximide chase assay [73]. Exponentially growing H1975 were treated with 3 µM KU60019 (Selleck Chem) for 48 h followed by 300 µg/mL cycloheximide (Sigma) and harvested at 0, 4, 8, 12 h post treatment. Protein extracts were analyzed by immunoblot assay and normalized to GAPDH levels.

H1975 lung cancer cells were treated with cycloheximide for increasing time in the presence of an ATM inhibitor (ATMi), KU 60019 [52], or vehicle, and cell extracts were analyzed for p53 and p (Ser15)-p53 levels.

### Immunoblot analysis and quantification

Immunoblot analysis was performed following standard techniques. Antibodies are described above. Quantitative comparisons were performed using Quantity One 4.6.2 software (Bio-Rad).

### siRNA transfection

To knockdown origin firing, Cdt1 and CDC7 siRNAs were obtained from Dharmacon as a pool of 4 individual siRNAs. A pool of non-targeting sicontrol (Dharmacon) was used as negative control. Cdt1 siRNA transfection was performed using Lipofectamine (Thermofisher) following manufacturer’s protocol. CDC7 siRNA transfection was performed using Cell Line NucleofectorTM KitV (Lonza) following manufacturer’s protocol.

### Calculation of combination index

Cells were seeded in 96-well plate and incubated with MK8776 and KU60019 with concentrations ranging from 10 µM to 1 nM. After 72 h, cell viability was determined using Alamar Blue assay (Thermofisher) following manufacturer’s protocol. Combination index was calculated using Chou-Talalay method as published [57].

### Xenograft studies

All animals used in this study were maintained and assayed in accordance with federal guidelines and those established by the Institutional Animal Care and Use Committee. NSG (NOD scid gamma) mice (VCU Cancer Mouse Model Core) were used for the tumorigenicity studies. Eight-week-old mice were injected with 1×10^7^ cells subcutaneously in both flanks and measured periodically following published protocols [69, 74]. H1975 cell line expressing shRNA against p53 or GFP was used. Once tumor was palpable, mice were equally distributed into treatment groups to receive 50 mg/kg of MK-8776 dissolved in 4% DMSO, 30% propylene glycol by intraperitoneal injection twice weekly, 10 mg/kg AZD0156 dissolved in 6% DMSO and 94% Captisol (30% w/v) orally daily, 5 mg/kg Chk2 inhibitor II hydrate dissolved in 4% DMSO, 30% propylene glycol by intraperitoneal injection daily, vehicle only or indicated combination of drugs. Once the tumors reached study end point, they were harvested for further analysis.

Lung orthotopic xenograft studies were performed by VCU mouse Core Laboratory. H1975 and A549 cells stably expressing luciferase (1 × 10^6^) were implanted orthotopically into the lung of NSG mouse. Tumor growth was monitored using in vivo imaging of luciferase expression every 7 days. Once tumors were detected mice were equally distributed into two groups: Control group (10% DMSO, 90% corn oil), and treated group (50 mg/kg of MK-8776, Chk1i, and 20 mg/kg AZD0156 dissolved in 10% DMSO, 90% corn oil). Treatment was administrated through oral or intra nasal injection every other day. Once the tumors reached study end point, they were harvested for further analysis.

### Immunohistochemistry

All staining were performed on formaldehyde fixed paraffin embedded (FFPE) sections. Samples were fixed in 4% paraformaldehyde for at least 24 h. Sections for TUNEL analysis were stained using Click-it Plus TUNEL assay kit (Thermofisher) following the company’s protocol. Fluorescent stained sections were mounted using ProLong Gold Anitfade with DAPI and imaged by confocal microscopy (Zeiss LSM700) at 40x magnification. Number of TUNEL positive cells were quantified using Image J software. For detection of p53 and chromosome segregation errors using patient-derived-xenograft and tumor cell line xenografts, tissues were fixed in 10% formalin for at least 24 h. Tissue embedding was performed by VCU Macromolecule Core Laboratory. Tissue sections (5 μm) were xylene deparaffinized and serially rehydrated in ethanol (100%, 95%, and 70%). Sections were treated with Antigen retrieval Tris/EDTA pH 9.0 buffer and blocked using SignalStain® Antibody Diluent (Cell Signaling #8112) for an hour, and incubated with anti-p53 (sc-123) antibody in a humidified chamber at 4 °C overnight. After 3 washes with PBST, slides were incubated with Alexafluor 594-conjugated rabbit anti-mouse for an hour. Fluorescent stained sections were mounted using ProLong Gold Anitfade with DAPI and imaged by confocal microscopy (Zeiss Cell Observer Spinning Disc confocal microscope) at ×60 magnification.

FFPE samples of H1975 tumor treated with Vehicle (10% DMSO + 90% corn oil) or ATM and Chk1 inhibitors were stained with anti-KI67 or anti-cleave-caspase 3 antibodies. A Vectra Polaris Automated microscope with 40X objective was used to image the whole stained slides and H&E slides, two all full scan per condition were analyzed from different mice. Whole slide scans were analyzed using QuPath software. Representative images show whole scan of the original H&E and stained images and markup for tumors stromal and normal tissue. Green indicates regions classified as stroma, dark red indicates tumor, blue represents normal lung tissue. QuPath software was also used to analyze percentage of positive Ki67 and c-caspase 3 cells in the tumor section. Two whole scan images per condition were used.

### TUNEL assay

FFPE tumor sections generated from mock-depleted (shGFP) and p53-depleted (shp53) H1975 xenografts after two consecutive treatments at 24 h interval with Chk1 (Chk1i) and/or ATM (ATMi) inhibitor or vehicle were immunostained for TUNEL assay. At least 10 blind images were taken and used for measurement. Analysis was repeated in three different mice tumor sections, representative data shown. Statistical analysis was performed using two-tailed Student’s *t* test.

### Whole slide Image analysis

A Vectra Polaris Automated microscope with 40X objective was used to image the whole slides after Hematoxylin & Eosin (H&E) staining and Ki67 and c-caspase-3 immunostaing. Whole slide scans were analyzed using QuPath software (GitHub, open-source). Representative images show whole scan of the original H&E and stained images and markup for tumors stromal and normal tissue.

### Statistics

Unless otherwise specified, all experiments were performed in triplicates. and mean ± SD data is shown. Presence of bi-directional origins and re-replicating origins generated by sequential labeling of replicating DNA in different samples in each experiment set was compared using two-sided Student’s *t* test. Swarm plots in each experimental set were compared using Mann–Whitney’s test. Bar graphs plotted as mean ± SD of three independent experiments, and were compared using two-sided Student’s *t* test. QPCR reactions were performed in duplicates and repeated in three independent experiments. All immunoblots have been repeated at least twice and representative data is shown. Unless otherwise specified for all the experiments a *p* value of less than 0.05 was considered significant and was calculated using GraphPad Prism8 software.

## Supplementary information

Supplemenrary Table S1

Supplementary Figures and Figure legends

Unedited blots

Time-lapse video image of mock depleted (shCT) H1048 lung cancer cells

Time-lapse video image of p53 depleted (shp53) H1048 lung cancer cells

Time-lapse video image of mock depleted (shCT) H1975 lung cancer cells

Time-lapse video image of p53 depleted (shp53) H1975 lung cancer cells

Time-lapse video image of H460 (Wt p53) lung cancer cells

Time-lapse video image of A549 (Wt p53) lung cancer cells

Time-lapse video image of mock depleted (shCT) H1975 lung cancer cells treated with DMSO

Time-lapse video image of mock depleted (shCT) H1975 lung cancer cells treated with ATM inhibitor (ATMi)

Time-lapse video image of mock depleted (shCT) H1975 lung cancer cells treated with Chk1 inhibitor (Chk1i)

Time-lapse video image of mock depleted (shCT) H1975 lung cancer cells treated with ATM and Chk1 inhibitors (ATMi+Chk1i)

Time-lapse video image of p53 depleted (shp53) H1975 lung cancer cells treated with DMSO

Time-lapse video image of p53 depleted (shp53) H1975 lung cancer cells treated with ATM inhibitor (ATMi)

Time-lapse video image of p53 depleted (shp53) H1975 lung cancer cells treated with Chk1 inhibitor (Chk1i)

Time-lapse video image of p53 depleted (shp53) H1975 lung cancer cells treated with ATM and Chk1 inhibitor (ATMi+Chk1i))

Time-lapse video image of WI38 normal human embryonic cells

## Data Availability

All data that support the findings of this study are available in the article and supplementary data. All images and scans of immunoblots are available upon request from the corresponding author.

## References

[1] p53 MUTATIONS IN LUNG CANCER - p53 WEB SITE. p53freefr/Database/p53_cancer/p53_Lunghtml.

[2] Soussi T, Wiman KG. TP53: an oncogene in disguise. Cell Death Differ. 2015;22:1239–49. 26024390 10.1038/cdd.2015.53 PMC4495363

[3] Bronte G, Rizzo S, La Paglia L, Adamo V, Siragusa S, Ficorella C, et al. Driver mutations and differential sensitivity to targeted therapies: a new approach to the treatment of lung adenocarcinoma. Cancer Treatt Rev. 2010;36:S21–9. 10.1016/S0305-7372(10)70016-5 21129606

[4] Vandin F, Upfal E, Raphael BJ. De novo discovery of mutated driver pathways in cancer. Genome Res. 2012;22:375–85. 21653252 10.1101/gr.120477.111 PMC3266044

[5] Hanel W, Marchenko N, Xu S, Yu SX, Weng W, Moll U. Two hot spot mutant p53 mouse models display differential gain of function in tumorigenesis. Cell Death Differ. 2013;20:898–909. 23538418 10.1038/cdd.2013.17 PMC3679454

[6] Olive KP, Tuveson DA, Ruhe ZC, Yin B, Willis NA, Bronson RT, et al. Mutant p53 gain of function in two mouse models of Li-Fraumeni syndrome. Cell. 2004;119:847–60. 15607980 10.1016/j.cell.2004.11.004

[7] Zheng S, El-Naggar AK, Kim ES, Kurie JM, Lozano G. A genetic mouse model for metastatic lung cancer with gender differences in survival. Oncogene. 2007;26:6896–904. 17486075 10.1038/sj.onc.1210493

[8] Oren M, Rotter V. Mutant p53 gain-of-function in cancer. Cold Spring Harb Perspect Biol. 2010;2:a001107. 20182618 10.1101/cshperspect.a001107 PMC2828285

[9] Vaughan CA, Frum R, Pearsall I, Singh S, Windle B, Yeudall A, et al. Allele specific gain-of-function activity of p53 mutants in lung cancer cells. Biochem Biophys Res Commun. 2012;428:6–10. 22989750 10.1016/j.bbrc.2012.09.029 PMC4731231

[10] Vaughan CA, Pearsall I, Singh S, Windle B, Deb SP, Grossman SR, et al. Addiction of lung cancer cells to GOF p53 is promoted by up-regulation of epidermal growth factor receptor through multiple contacts with p53 transactivation domain and promoter. Oncotarget. 2016;7:12426–46. 26820293 10.18632/oncotarget.6998 PMC4914296

[11] Vaughan CA, Singh S, Windle B, Sankala HM, Graves PR, Andrew Yeudall W, et al. p53 mutants induce transcription of NF-kappaB2 in H1299 cells through CBP and STAT binding on the NF-kappaB2 promoter and gain of function activity. Arch Biochem Biophys. 2011;518:79–88. 22198284 10.1016/j.abb.2011.12.006 PMC3272778

[12] Alexandrova EM, Yallowitz AR, Li D, Xu S, Schulz R, Proia DA, et al. Improving survival by exploiting tumour dependence on stabilized mutant p53 for treatment. Nature. 2015;523:352–56. 26009011 10.1038/nature14430 PMC4506213

[13] Schulz-Heddergott R, Stark N, Edmunds SJ, Li J, Conradi LC, Bohnenberger H, et al. Therapeutic ablation of gain-of-function mutant p53 in colorectal cancer inhibits Stat3-mediated tumor growth and invasion. Cancer Cell. 2018;34:298–314. 30107178 10.1016/j.ccell.2018.07.004 PMC6582949

[14] Singh S, Vaughan CA, Frum RA, Grossman SR, Deb S, Palit Deb S. Mutant p53 establishes targetable tumor dependency by promoting unscheduled replication. J Clin Invest. 2017;127:1839–55. 28394262 10.1172/JCI87724 PMC5409068

[15] Freed-Pastor WA, Prives C. Mutant p53: one name, many proteins. Genes Dev. 2012;26:1268–86. 22713868 10.1101/gad.190678.112 PMC3387655

[16] Vaughan CA, Singh S, Grossman SR, Windle B, Deb SP, Deb S. Gain-of-function p53 activates multiple signaling pathways to induce oncogenicity in lung cancer cells. Mol Oncol. 2017;11:696–711. 28423230 10.1002/1878-0261.12068 PMC5467493

[17] Zhu J, Sammons MA, Donahue G, Dou Z, Vedadi M, Getlik M, et al. Gain-of-function p53 mutants co-opt chromatin pathways to drive cancer growth. Nature. 2015;525:206–11. 26331536 10.1038/nature15251 PMC4568559

[18] Vaughan CA, Singh S, Subler MA, Windle JJ, Inoue K, Fry EA, et al. The oncogenicity of tumor-derived mutant p53 is enhanced by the recruitment of PLK3. Nat Commun. 2021;12:704. 33514736 10.1038/s41467-021-20928-8 PMC7846773

[19] Neelsen KJ, Zanini IM, Mijic S, Herrador R, Zellweger R, Ray Chaudhuri A, et al. Deregulated origin licensing leads to chromosomal breaks by rereplication of a gapped DNA template. Genes Dev. 2013;27:2537–42. 24298053 10.1101/gad.226373.113 PMC3861667

[20] Bartek J, Bartkova J, Lukas J. DNA damage signalling guards against activated oncogenes and tumour progression. Oncogene. 2007;26:7773–9. 18066090 10.1038/sj.onc.1210881

[21] Fragkos M, Naim V. Rescue from replication stress during mitosis. Cell Cycle. 2017;16:613–33. 28166452 10.1080/15384101.2017.1288322 PMC5397263

[22] Hsieh HJ, Peng G. Cellular responses to replication stress: Implications in cancer biology and therapy. DNA Repair (Amst). 2017;49:9–20. 27908669 10.1016/j.dnarep.2016.11.002

[23] Schmidt V, Nagar R, Martinez LA. Control of Nucleotide Metabolism Enables Mutant p53’s Oncogenic Gain-of-Function Activity. Int J Mol Sci. 2017;18:2759. 29257071 10.3390/ijms18122759 PMC5751358

[24] Siegel JJ, Amon A. New insights into the troubles of aneuploidy. Annu Rev Cell Dev Biol. 2012;28:189–214. 22804579 10.1146/annurev-cellbio-101011-155807 PMC3919630

[25] Cimini D. Twenty years of merotelic kinetochore attachments: a historical perspective. Chromosome Res. 2023;31:18. 37466740 10.1007/s10577-023-09727-7 PMC10411636

[26] Fujiwara T, Bandi M, Nitta M, Ivanova EV, Bronson RT, Pellman D. Cytokinesis failure generating tetraploids promotes tumorigenesis in p53-null cells. Nature. 2005;437:1043–7. 16222300 10.1038/nature04217

[27] Lens SMA, Medema RH. Cytokinesis defects and cancer. Nat Rev Cancer. 2019;19:32–45. 30523339 10.1038/s41568-018-0084-6

[28] Cortez D. Preventing replication fork collapse to maintain genome integrity. DNA repair (Amst). 2015;32:149–57. 25957489 10.1016/j.dnarep.2015.04.026 PMC4522347

[29] McIntosh D, Blow JJ. Dormant origins, the licensing checkpoint, and the response to replicative stresses. Cold Spring Harb Perspect Biol. 2012;4:a012955. 22904560 10.1101/cshperspect.a012955 PMC3475168

[30] Frum RA, Deb S, Deb SP. Use of the DNA fiber spreading technique to detect the effects of mutant p53 on DNA replication. Methods Mol Biol. 2013;962:147–55. 23150444 10.1007/978-1-62703-236-0_12 PMC4839281

[31] Frum RA, Singh S, Vaughan C, Mukhopadhyay ND, Grossman SR, Windle B, et al. The human oncoprotein MDM2 induces replication stress eliciting early intra-S-phase checkpoint response and inhibition of DNA replication origin firing. Nucleic Acids Res. 2014;42:926–40. 24163099 10.1093/nar/gkt944 PMC3902934

[32] Graham JE, Marians KJ, Kowalczykowski SC. Independent and Stochastic Action of DNA Polymerases in the Replisome. Cell. 2017;169:1201–13. 28622507 10.1016/j.cell.2017.05.041 PMC5548433

[33] Dorn ES, Chastain PD 2nd, Hall JR, Cook JG. Analysis of re-replication from deregulated origin licensing by DNA fiber spreading. Nucleic Acids Res. 2009;37:60–9. 19010964 10.1093/nar/gkn912 PMC2615611

[34] Neelsen KJ, Zanini IM, Herrador R, Lopes M. Oncogenes induce genotoxic stress by mitotic processing of unusual replication intermediates. J Cell Biol. 2013;200:699–708. 23479741 10.1083/jcb.201212058 PMC3601361

[35] Lukas C, Savic V, Bekker-Jensen S, Doil C, Neumann B, Pedersen RS, et al. 53BP1 nuclear bodies form around DNA lesions generated by mitotic transmission of chromosomes under replication stress. Nat Cell Biol. 2011;13:243–53. 21317883 10.1038/ncb2201

[36] Pellegrino S, Michelena J, Teloni F, Imhof R, Altmeyer M. Replication-Coupled Dilution of H4K20me2 Guides 53BP1 to Pre-replicative Chromatin. Cell Rep. 2017;19:1819–31. 28564601 10.1016/j.celrep.2017.05.016 PMC5857200

[37] Escribano-Diaz C, Orthwein A, Fradet-Turcotte A, Xing M, Young JT, Tkac J, et al. A cell cycle-dependent regulatory circuit composed of 53BP1-RIF1 and BRCA1-CtIP controls DNA repair pathway choice. Mol Cell. 2013;49:872–83. 23333306 10.1016/j.molcel.2013.01.001

[38] Fernandez-Vidal A, Vignard J, Mirey G. Around and beyond 53BP1 Nuclear Bodies. Int J Mol Sci. 2017;18:2611. 29206178 10.3390/ijms18122611 PMC5751214

[39] Bakkenist CJ, Kastan MB. Chromatin perturbations during the DNA damage response in higher eukaryotes. DNA repair (Amst). 2015;36:8–12. 26391293 10.1016/j.dnarep.2015.09.002 PMC4727245

[40] Bartek J, Falck J, Lukas J. CHK2 kinase-a busy messenger. Nat Rev Mol Cell Biol. 2001;2:877–86. 11733767 10.1038/35103059

[41] Ratnayeke N, Baris Y, Chung M, Yeeles JTP, Meyer T. CDT1 inhibits CMG helicase in early S phase to separate origin licensing from DNA synthesis. Mol Cell. 2023;83:26–42. 36608667 10.1016/j.molcel.2022.12.004 PMC7614657

[42] Varma D, Chandrasekaran S, Sundin LJ, Reidy KT, Wan X, Chasse DA, et al. Recruitment of the human Cdt1 replication licensing protein by the loop domain of Hec1 is required for stable kinetochore-microtubule attachment. Nat Cell Biol. 2012;14:593–603. 22581055 10.1038/ncb2489 PMC3366049

[43] Petropoulos M, Champeris Tsaniras S, Taraviras S, Lygerou Z. Replication Licensing Aberrations, Replication Stress, and Genomic Instability. Trends Biochem Sci. 2019;44:752–64. 31054805 10.1016/j.tibs.2019.03.011

[44] Marques JF, Kops G. Permission to pass: on the role of p53 as a gatekeeper for aneuploidy. Chromosome Res. 2023;31:31. 37864038 10.1007/s10577-023-09741-9 PMC10589155

[45] Overholtzer M, Rao PH, Favis R, Lu XY, Elowitz MB, Barany F, et al. The presence of p53 mutations in human osteosarcomas correlates with high levels of genomic instability. Proc Natl Acad Sci USA. 2003;100:11547–52. 12972634 10.1073/pnas.1934852100 PMC208795

[46] Baronio R, Danziger SA, Hall LV, Salmon K, Hatfield GW, Lathrop RH, et al. All-codon scanning identifies p53 cancer rescue mutations. Nucleic Acids Res. 2010;38:7079–88. 20581117 10.1093/nar/gkq571 PMC2978351

[47] Barta JA, Pauley K, Kossenkov AV, McMahon SB. The lung-enriched p53 mutants V157F and R158L/P regulate a gain of function transcriptome in lung cancer. Carcinogenesis. 2020;41:67–77. 31067569 10.1093/carcin/bgz087 PMC7316406

[48] Vijayakumaran R, Tan KH, Miranda PJ, Haupt S, Haupt Y. Regulation of mutant p53 protein expression. Front Oncol. 2015;5:284. 26734569 10.3389/fonc.2015.00284 PMC4681805

[49] Zhou X, Hao Q, Lu H. Mutant p53 in cancer therapy-the barrier or the path. J Mol Cell Biol. 2019;11:293–305. 30508182 10.1093/jmcb/mjy072 PMC6487791

[50] Bartek J, Bartkova J, Vojtesek B, Staskova Z, Lukas J, Rejthar A, et al. Aberrant expression of the p53 oncoprotein is a common feature of a wide spectrum of human malignancies. Oncogene. 1991;6:1699–703. 1923535

[51] Reihsaus E, Kohler M, Kraiss S, Oren M, Montenarh M. Regulation of the level of the oncoprotein p53 in non-transformed and transformed cells. Oncogene. 1990;5:137–45. 2157179

[52] Golding SE, Rosenberg E, Valerie N, Hussaini I, Frigerio M, Cockcroft XF, et al. Improved ATM kinase inhibitor KU-60019 radiosensitizes glioma cells, compromises insulin, AKT and ERK prosurvival signaling, and inhibits migration and invasion. Mol Cancer Ther. 2009;8:2894–902. 19808981 10.1158/1535-7163.MCT-09-0519 PMC2761990

[53] Ennis HL, Lubin M. Cycloheximide: aspects of inhibition of protein synthesis in mammalian cells. Science. 1964;146:1474–6. 14208575 10.1126/science.146.3650.1474

[54] Lopes M, Cotta-Ramusino C, Pellicioli A, Liberi G, Plevani P, Muzi-Falconi M, et al. The DNA replication checkpoint response stabilizes stalled replication forks. Nature. 2001;412:557–61. 11484058 10.1038/35087613

[55] Guzi TJ, Paruch K, Dwyer MP, Labroli M, Shanahan F, Davis N, et al. Targeting the replication checkpoint using SCH 900776, a potent and functionally selective CHK1 inhibitor identified via high content screening. Mol Cancer Ther. 2011;10:591–602. 21321066 10.1158/1535-7163.MCT-10-0928

[56] Mackay DR, Ullman KS. ATR and a Chk1-Aurora B pathway coordinate postmitotic genome surveillance with cytokinetic abscission. Mol Biol Cell. 2015;26:2217–26. 25904336 10.1091/mbc.E14-11-1563 PMC4462940

[57] Chou TC. Drug combination studies and their synergy quantification using the Chou-Talalay method. Cancer Res. 2010;70:440–6. 20068163 10.1158/0008-5472.CAN-09-1947

[58] Pagliarini R, Shao W, Sellers WR. Oncogene addiction: pathways of therapeutic response, resistance, and road maps toward a cure. EMBO Rep. 2015;16:280–96. 25680965 10.15252/embr.201439949 PMC4364868

[59] Vaughan CA, Deb SP, Deb S, Windle B. Preferred binding of gain-of-function mutant p53 to bidirectional promoters with coordinated binding of ETS1 and GABPA to multiple binding sites. Oncotarget. 2014;5:417–27. 24481480 10.18632/oncotarget.1708 PMC3964217

[60] Wang Z, Burigotto M, Ghetti S, Vaillant F, Tan T, Capaldo BD, et al. Loss-of-Function but Not Gain-of-Function Properties of Mutant TP53 Are Critical for the Proliferation, Survival, and Metastasis of a Broad Range of Cancer Cells. Cancer Discov. 2024;14:362–79. 37877779 10.1158/2159-8290.CD-23-0402 PMC10850947

[61] Zhao M, Wang T, Gleber-Netto FO, Chen Z, McGrail DJ, Gomez JA, et al. Mutant p53 gains oncogenic functions through a chromosomal instability-induced cytosolic DNA response. Nat Commun. 2024;15:180. 38167338 10.1038/s41467-023-44239-2 PMC10761733

[62] Jaber S, Eldawra E, Rakotopare J, Simeonova I, Lejour V, Gabriel M, et al. Oncogenic and teratogenic effects of Trp53(Y217C), an inflammation-prone mouse model of the human hotspot mutant TP53(Y220C). Elife. 2025;13:RP102434. 10.7554/eLife.102434 PMC11996178 40223808

[63] Maya-Mendoza A, Ostrakova J, Kosar M, Hall A, Duskova P, Mistrik M, et al. Myc and Ras oncogenes engage different energy metabolism programs and evoke distinct patterns of oxidative and DNA replication stress. Mol Oncol. 2015;9:601–16. 25435281 10.1016/j.molonc.2014.11.001 PMC5528704

[64] Song H, Hollstein M, Xu Y. p53 gain-of-function cancer mutants induce genetic instability by inactivating ATM. Nat Cell Biol. 2007;9:573–80. 17417627 10.1038/ncb1571

[65] Li D, Marchenko ND, Schulz R, Fischer V, Velasco-Hernandez T, Talos F, et al. Functional inactivation of endogenous MDM2 and CHIP by HSP90 causes aberrant stabilization of mutant p53 in human cancer cells. Mol Cancer Res. 2011;9:577–88. 21478269 10.1158/1541-7786.MCR-10-0534 PMC3097033

[66] Chougoni KK, Neely V, Ding B, Oduah E, Lam VT, Hu B, et al. Oncogenic Mutant p53 Sensitizes Non-Small Cell Lung Cancer Cells to Proteasome Inhibition via Oxidative Stress-Dependent Induction of Mitochondrial Apoptosis. Cancer Res Commun. 2024;4:2685–98. 39302104 10.1158/2767-9764.CRC-23-0637 PMC11474859

[67] Montagnoli A, Valsasina B, Croci V, Menichincheri M, Rainoldi S, Marchesi V, et al. A Cdc7 kinase inhibitor restricts initiation of DNA replication and has antitumor activity. Nat Chem Biol. 2008;4:357–65. 18469809 10.1038/nchembio.90

[68] Dohmen AJC, Qiao X, Duursma A, Wijdeven RH, Lieftink C, Hageman F, et al. Identification of a novel ATM inhibitor with cancer cell specific radiosensitization activity. Oncotarget. 2017;8:73925–37. 29088757 10.18632/oncotarget.18034 PMC5650312

[69] Suzuki M, Yamamori T, Bo T, Sakai Y, Inanami O. MK-8776, a novel Chk1 inhibitor, exhibits an improved radiosensitizing effect compared to UCN-01 by exacerbating radiation-induced aberrant mitosis. Transl Oncol. 2017;10:491–500. 28550769 10.1016/j.tranon.2017.04.002 PMC5447387

[70] Dai B, Zhao XF, Mazan-Mamczarz K, Hagner P, Corl S, Bahassi el M, et al. Functional and molecular interactions between ERK and CHK2 in diffuse large B-cell lymphoma. Nat Commun. 2011;2:402. 21772273 10.1038/ncomms1404 PMC3144586

[71] Mukherjee B, Tomimatsu N, Burma S. Immunofluorescence-based methods to monitor DNA end resection. Methods Mol Biol (Clifton, NJ). 2015;1292:67–75. 10.1007/978-1-4939-2522-3_5 PMC5482411 25804748

[72] Scian MJ, Stagliano KE, Deb D, Ellis MA, Carchman EH, Das A, et al. Tumor-derived p53 mutants induce oncogenesis by transactivating growth-promoting genes. Oncogene. 2004;23:4430–43. 15077194 10.1038/sj.onc.1207553

[73] Kao SH, Wang WL, Chen CY, Chang YL, Wu YY, Wang YT, et al. Analysis of protein stability by the cycloheximide chase assay. Bio Protoc. 2015;5:e1374. 29082276 10.21769/BioProtoc.1374 PMC5659619

[74] Pike KG, Barlaam B, Cadogan E, Campbell A, Chen Y, Colclough N, et al. The Identification of Potent, Selective, and Orally Available Inhibitors of Ataxia Telangiectasia Mutated (ATM) Kinase: The Discovery of AZD0156 (8-{6-[3-(Dimethylamino)propoxy]pyridin-3-yl}-3-methyl-1-(tetrahydro-2 H-pyran-4-yl)-1,3-dihydro-2 H-imidazo[4,5- c]quinolin-2-one). J Med Chem. 2018;61:3823–41. 29683659 10.1021/acs.jmedchem.7b01896

