# Supplementary material for: Oncogenic p53 induces mitotic errors in lung cancer cells by recopying DNA replication forks conferring targetable proliferation advantage
Source: Cell Death Differ. 2026 Jan 26;33(8):1585–97. doi: 10.1038/s41418-026-01670-4 (PMC13433782; doi:10.1038/s41418-026-01670-4)
Supplement: Supplementary file 1 [file 41418_2026_1670_MOESM3_ESM.pdf]

Fig. 1A

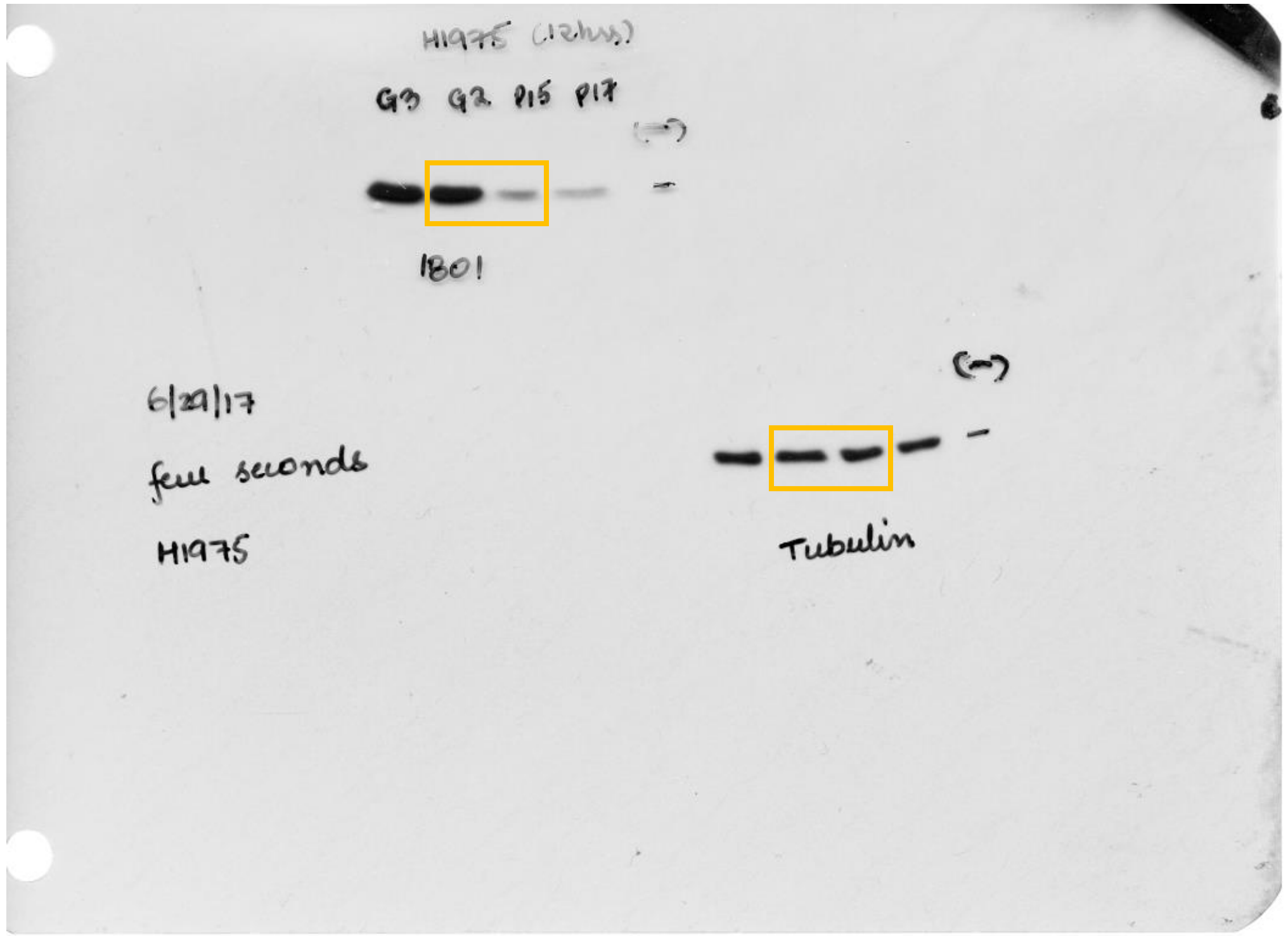
H1975 (12hrs)
G3 G2 P15 P17 (-)
1801
6/29/17
few seconds
H1975
(-)
Tubulin

Fig. 2D

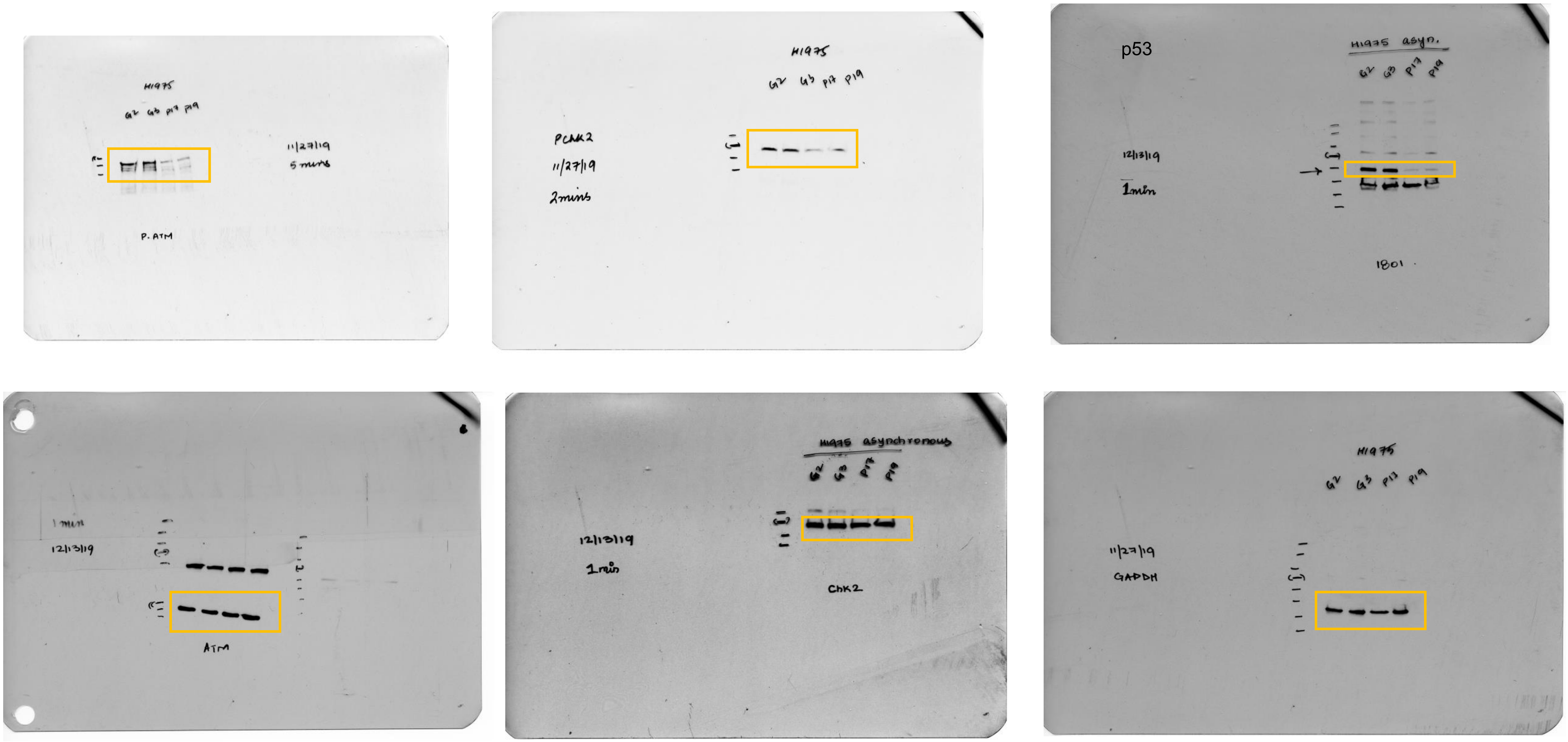

H1975
G2 G3 p17 p19
11/27/19
5 mins
P. ATM
H1975
G2 G3 p17 p19
PChk2
11/27/19
2mins
p53
H1975 asyn.
G2 G3 p17 p19
12/13/19
1min
1801
1 min
12/13/19
ATM
H1975 asynchronous
G2 G3 p17 p19
12/13/19
1min
Chk2
H1975
G2 G3 p13 p19
11/27/19
GAPDH

Fig. 3A

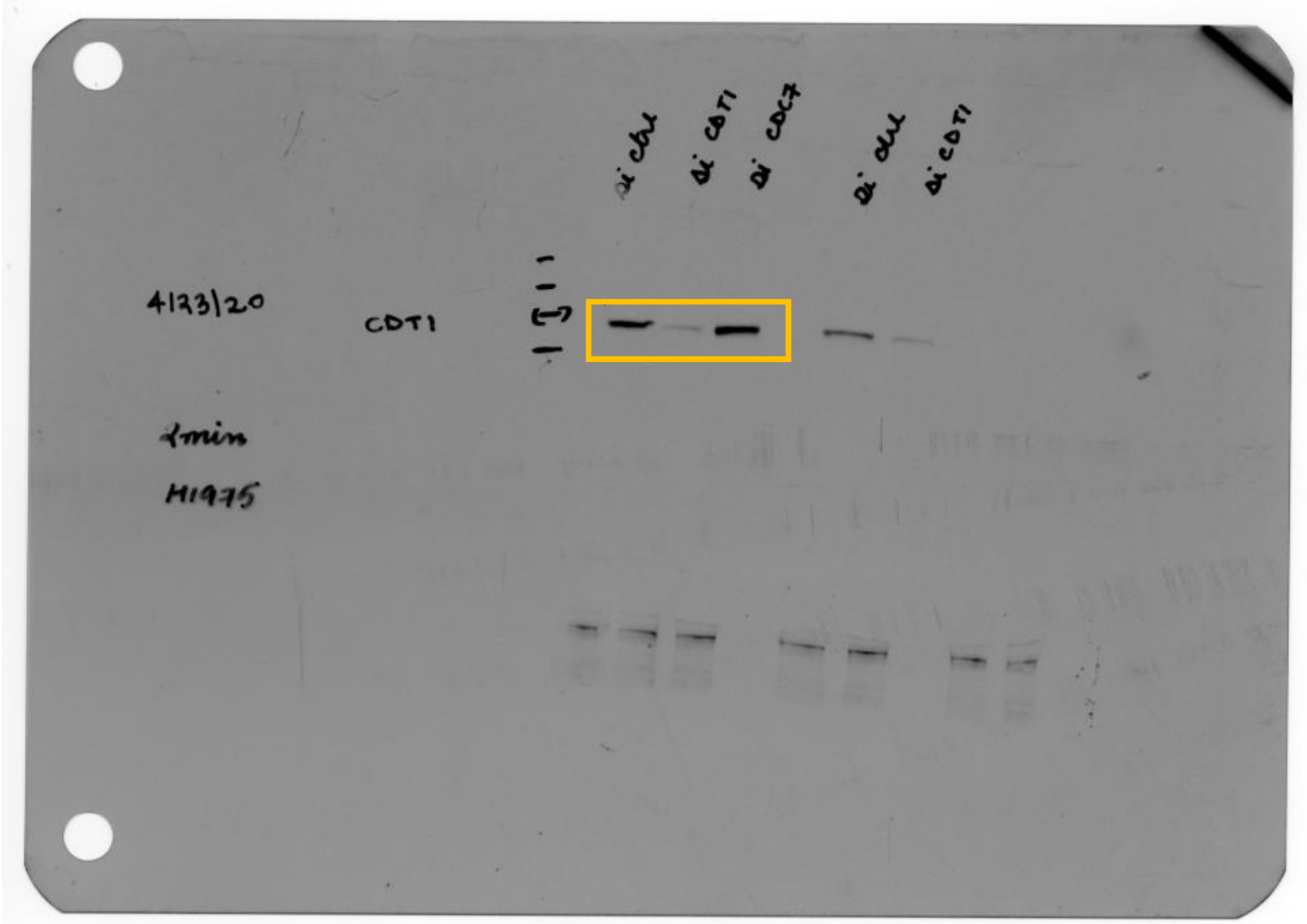

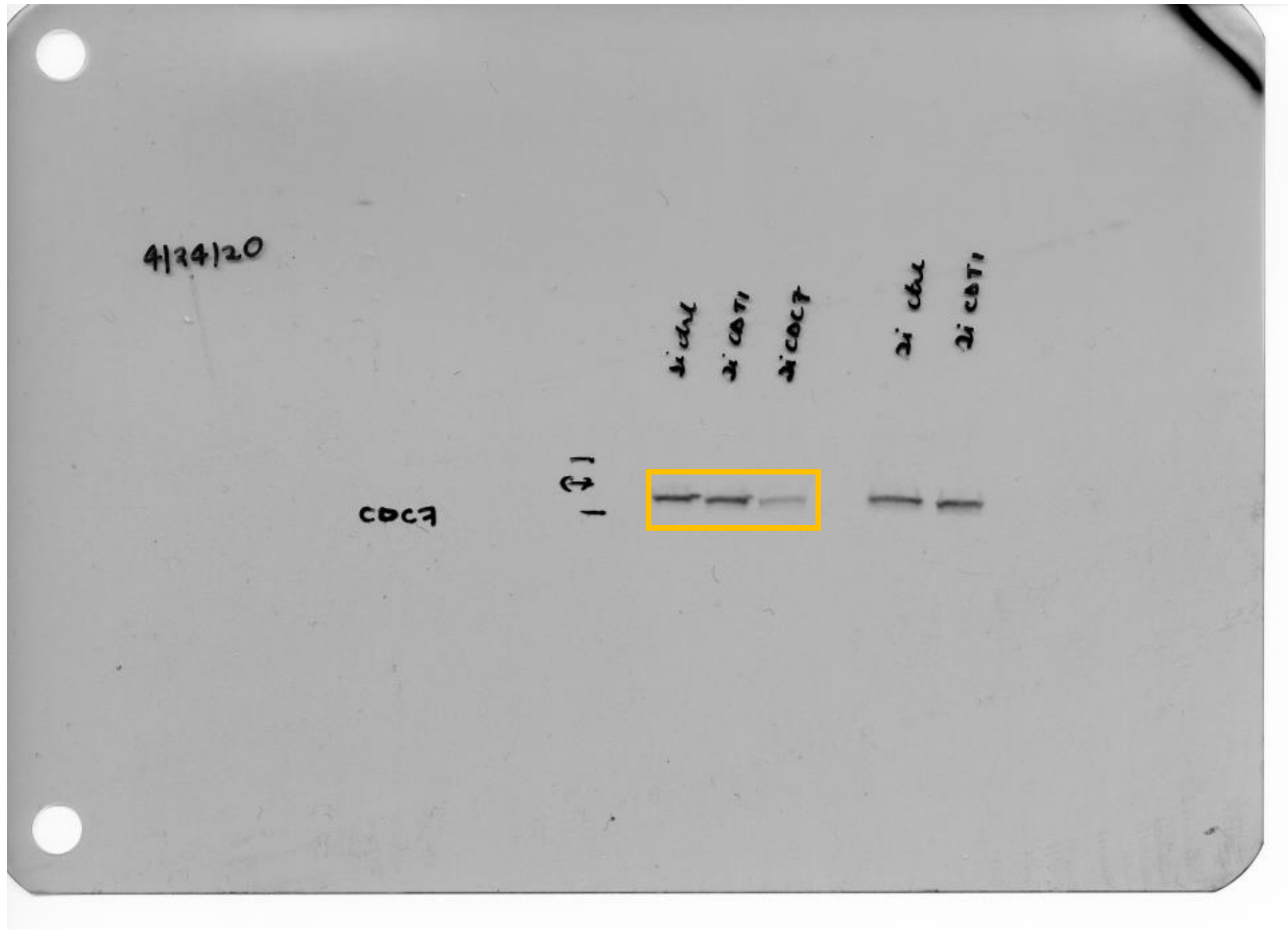

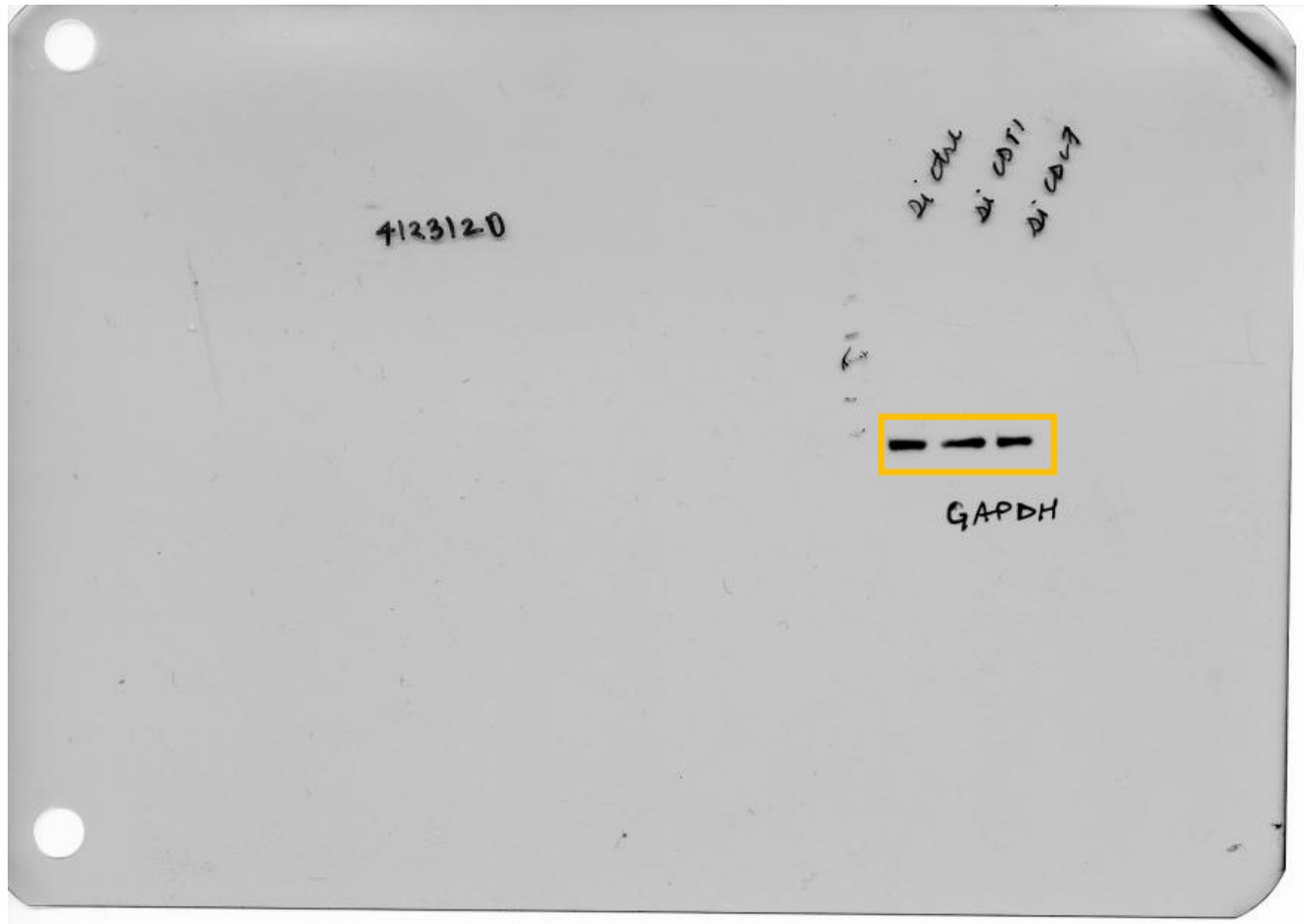

Fig. 4D

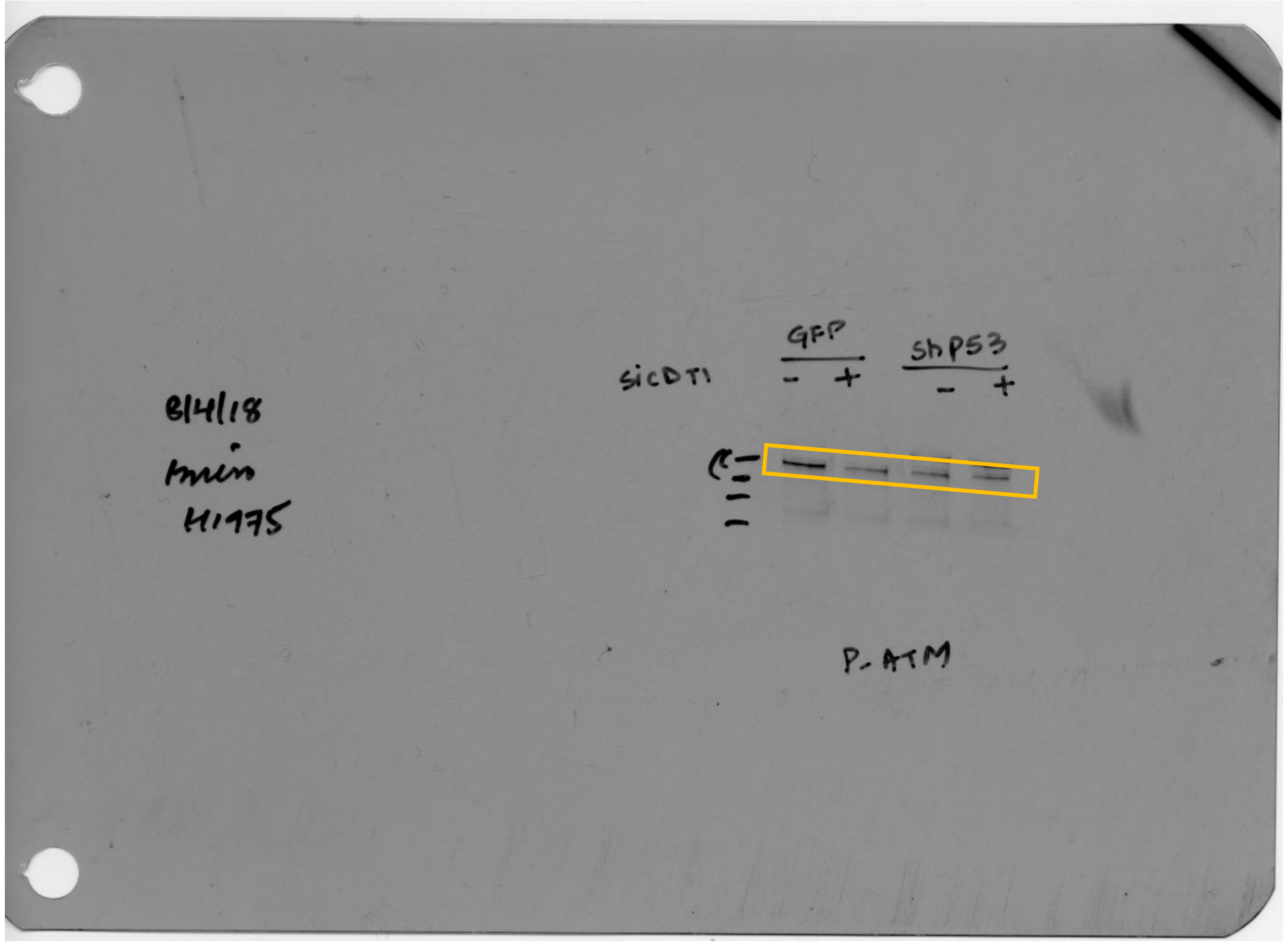

Cdt1

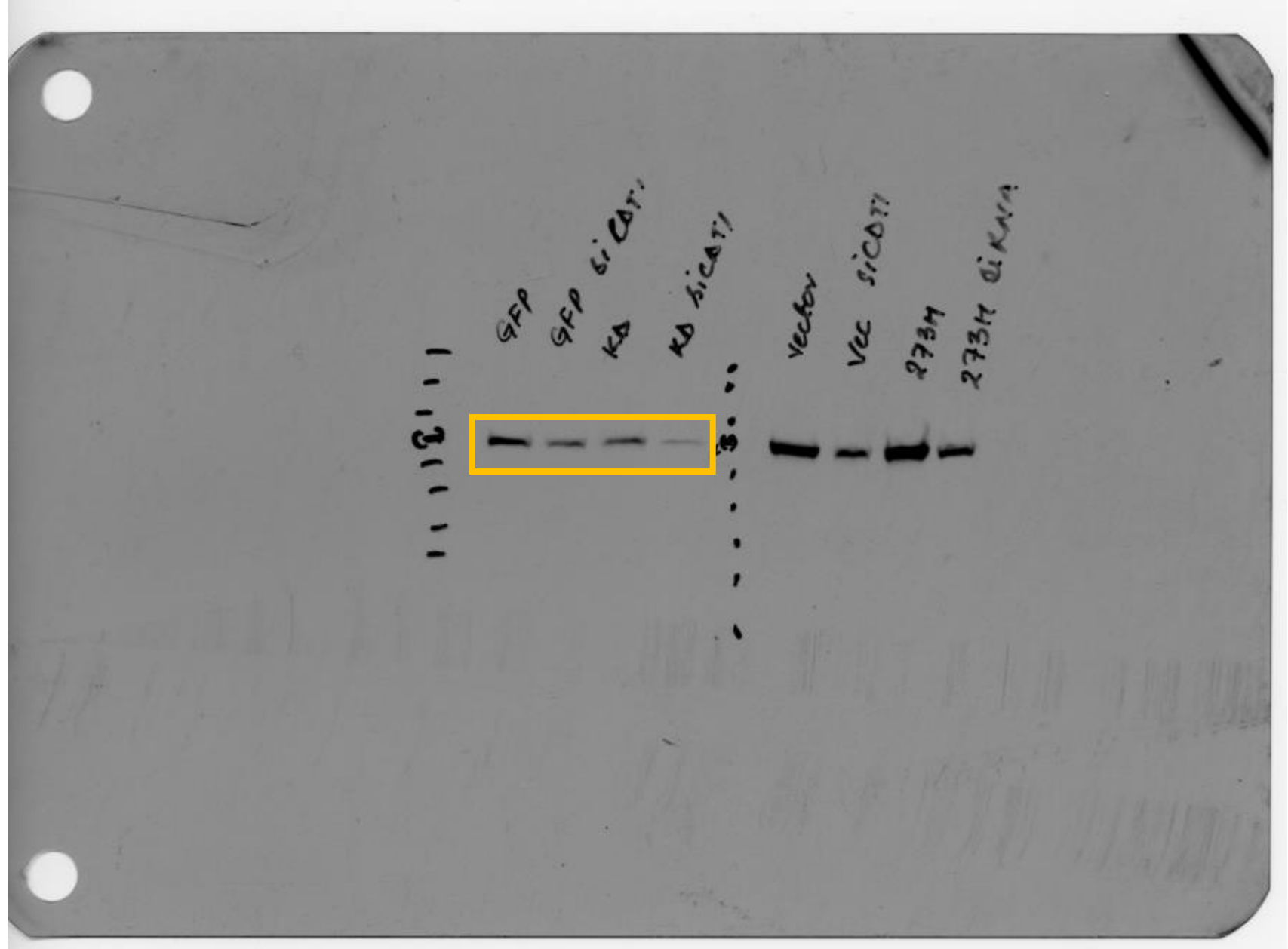

ATM

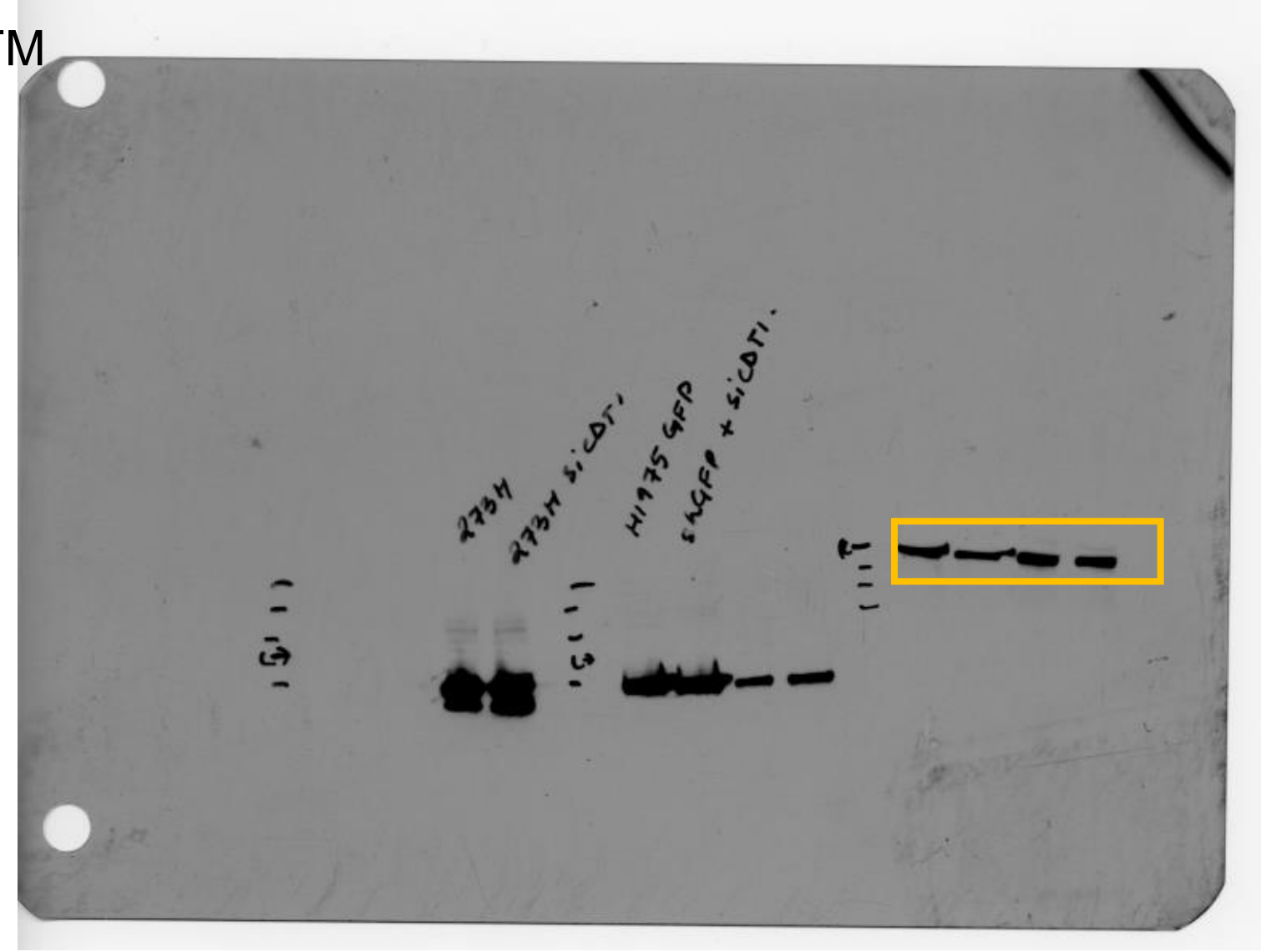

Fig. 4D

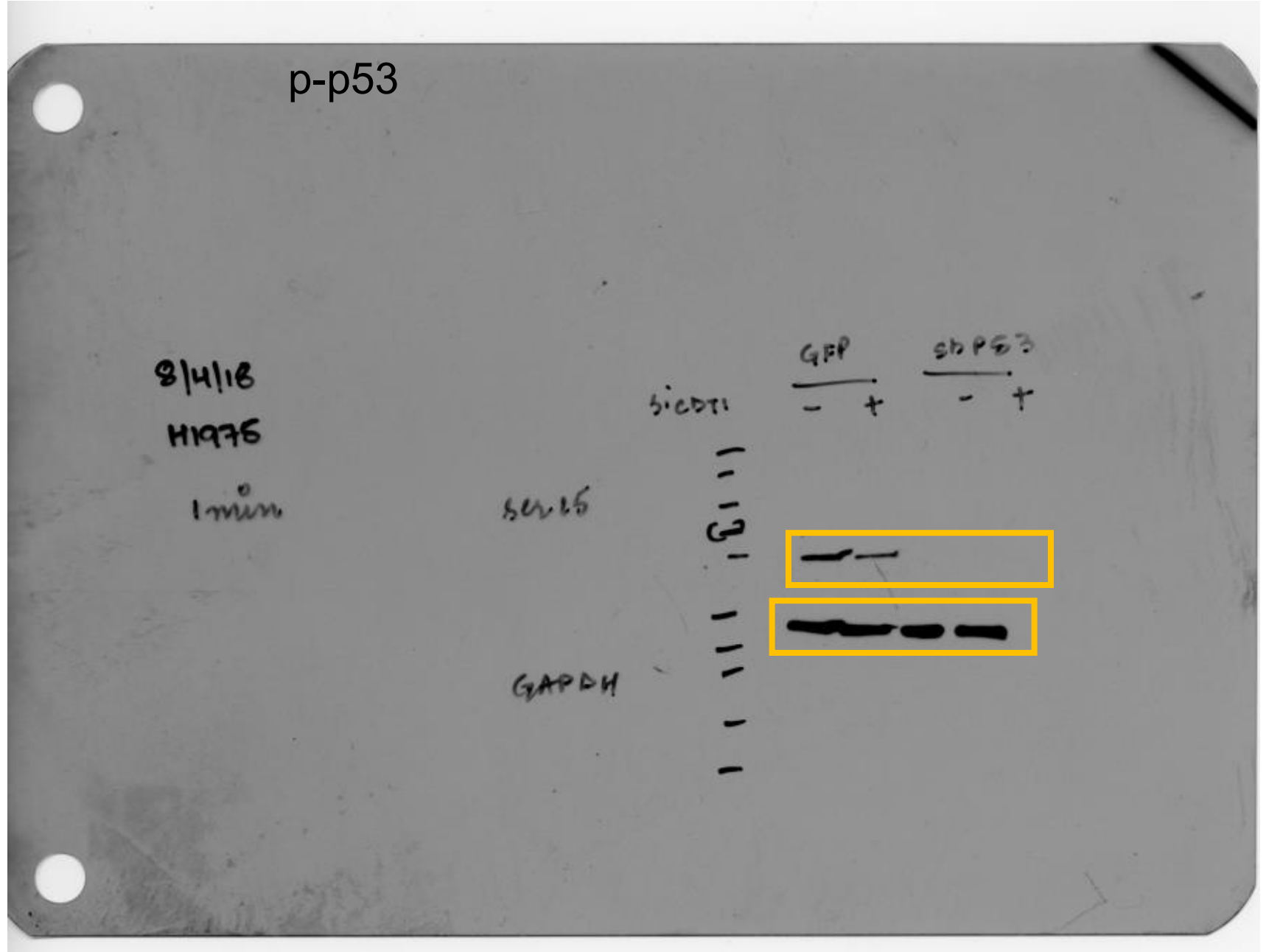
p-p53
8/4/18
H1975
GFP
shp53
- +
- +
GAPDH

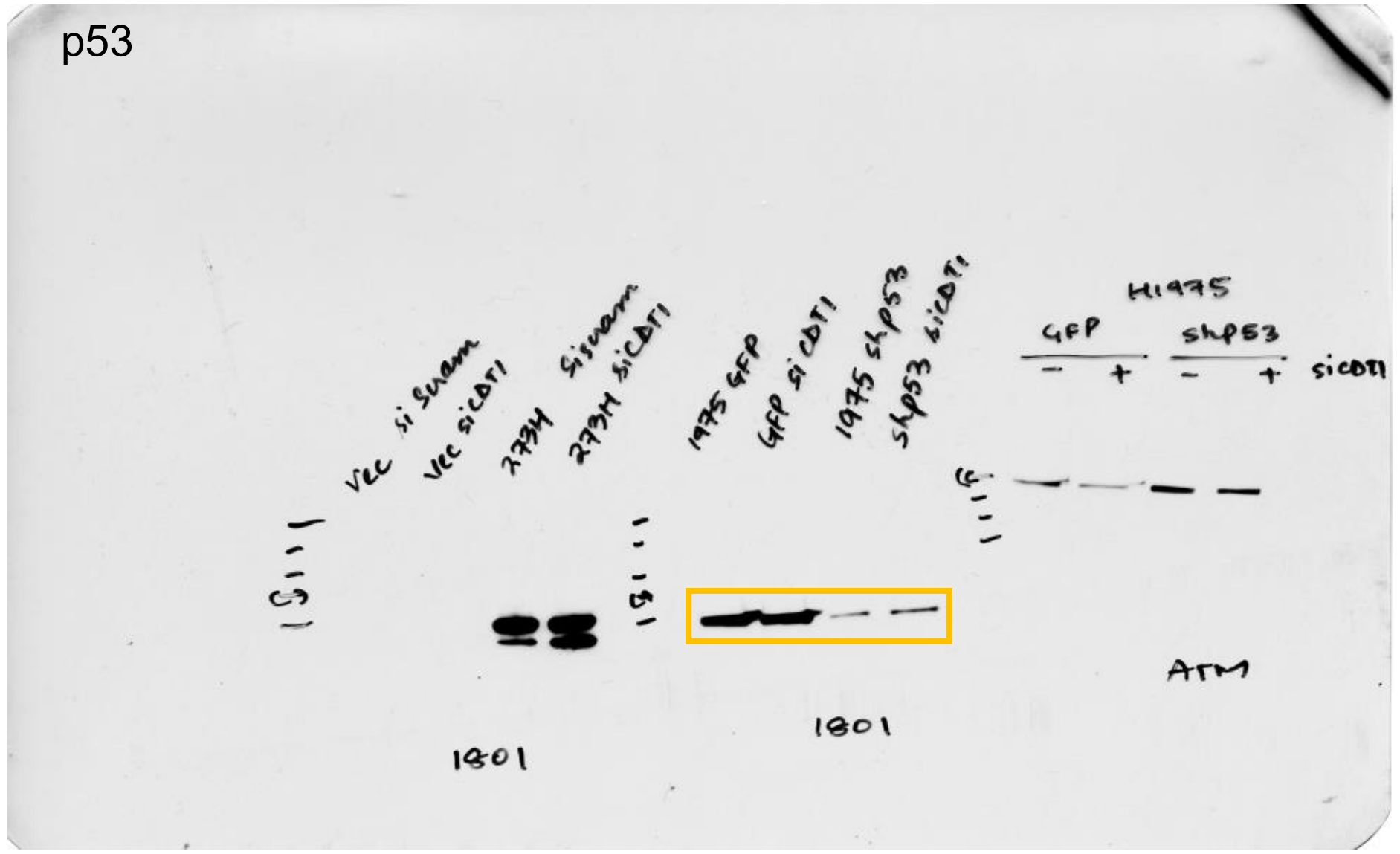
p53
H1975
GFP
shp53
- +
- +
1801
1801
ATM

Fig. 4E

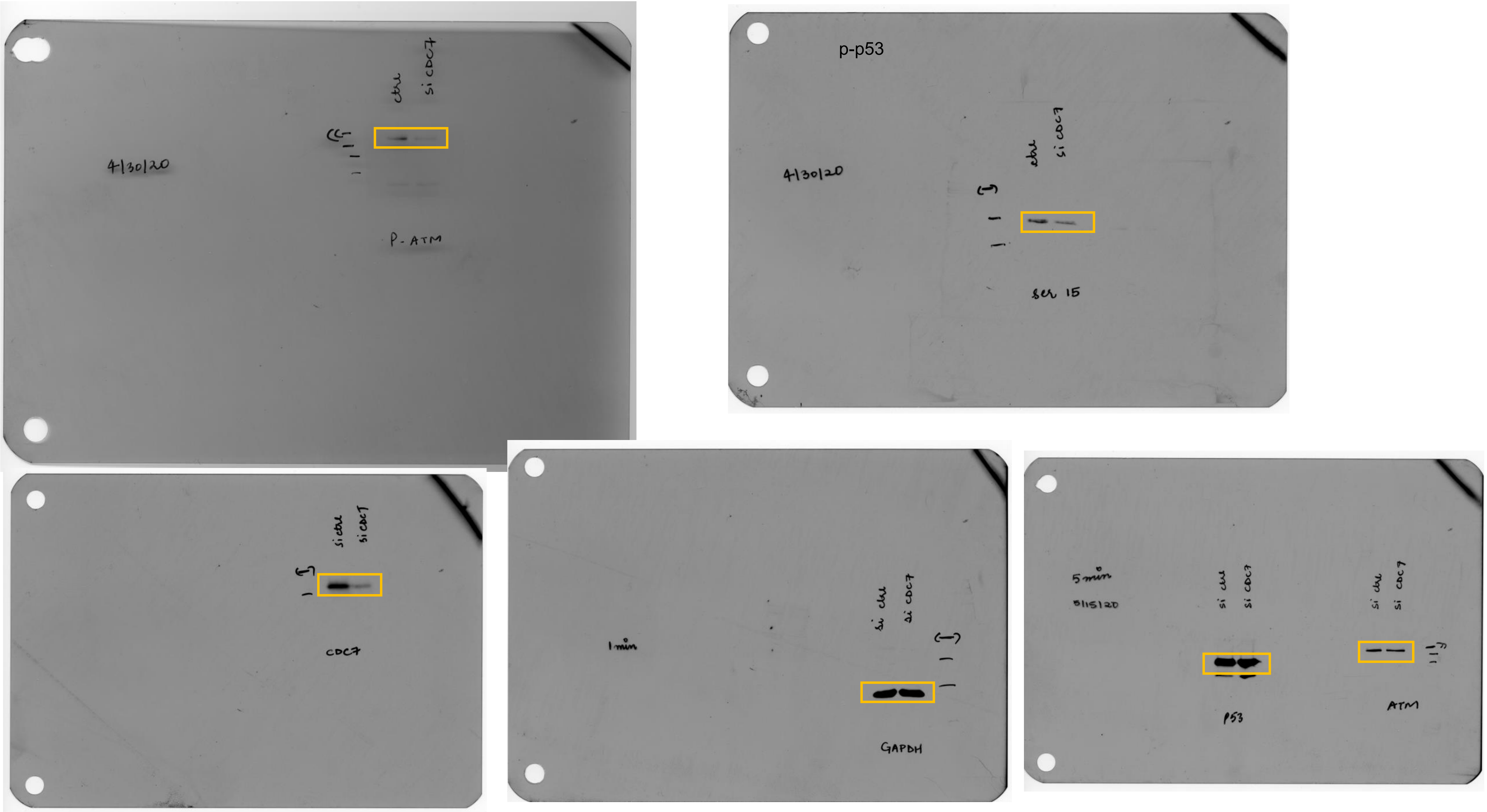

Fig. 6A

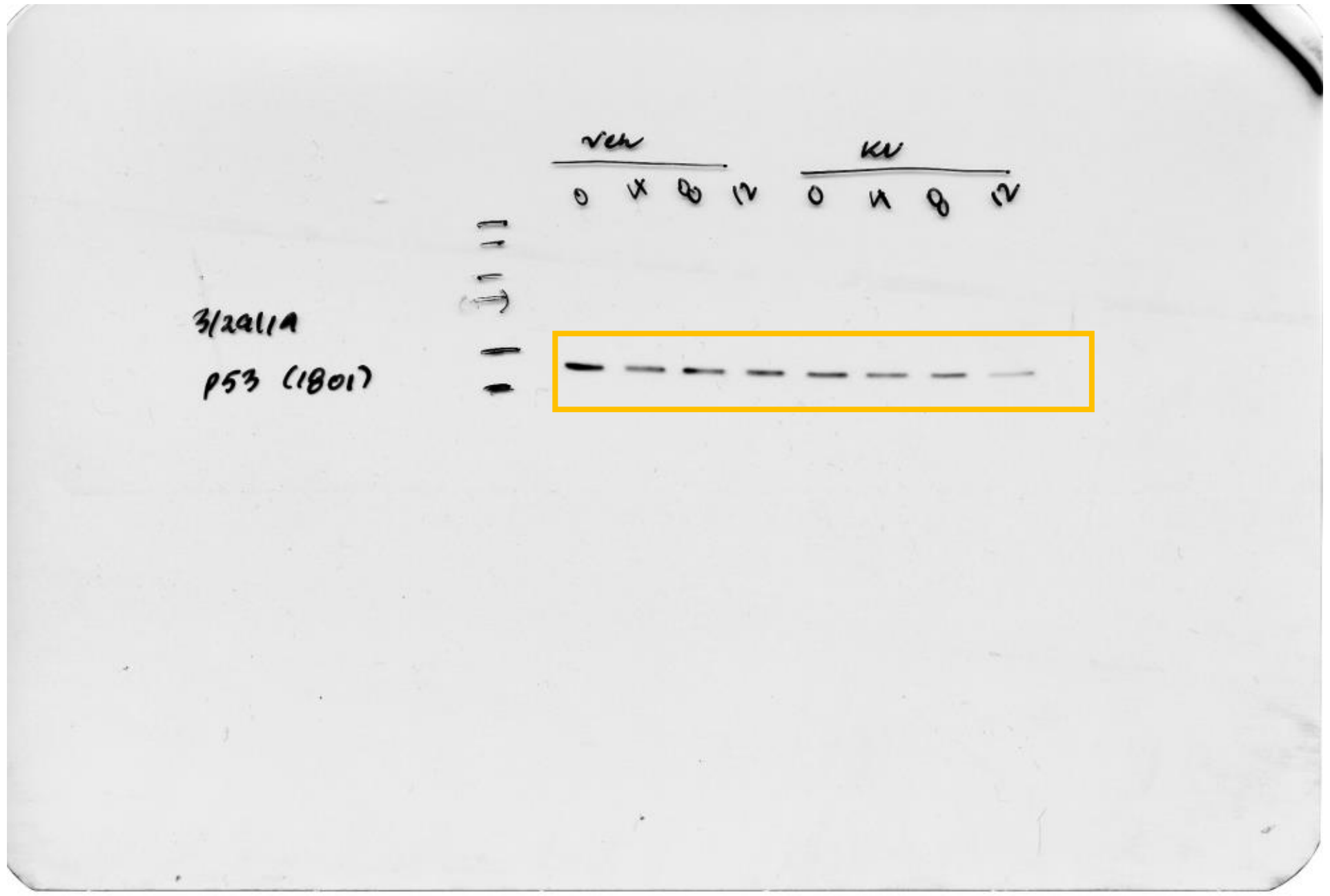
veh
KU
0 4 8 12 0 4 8 12
3/29/19
p53 (1801)

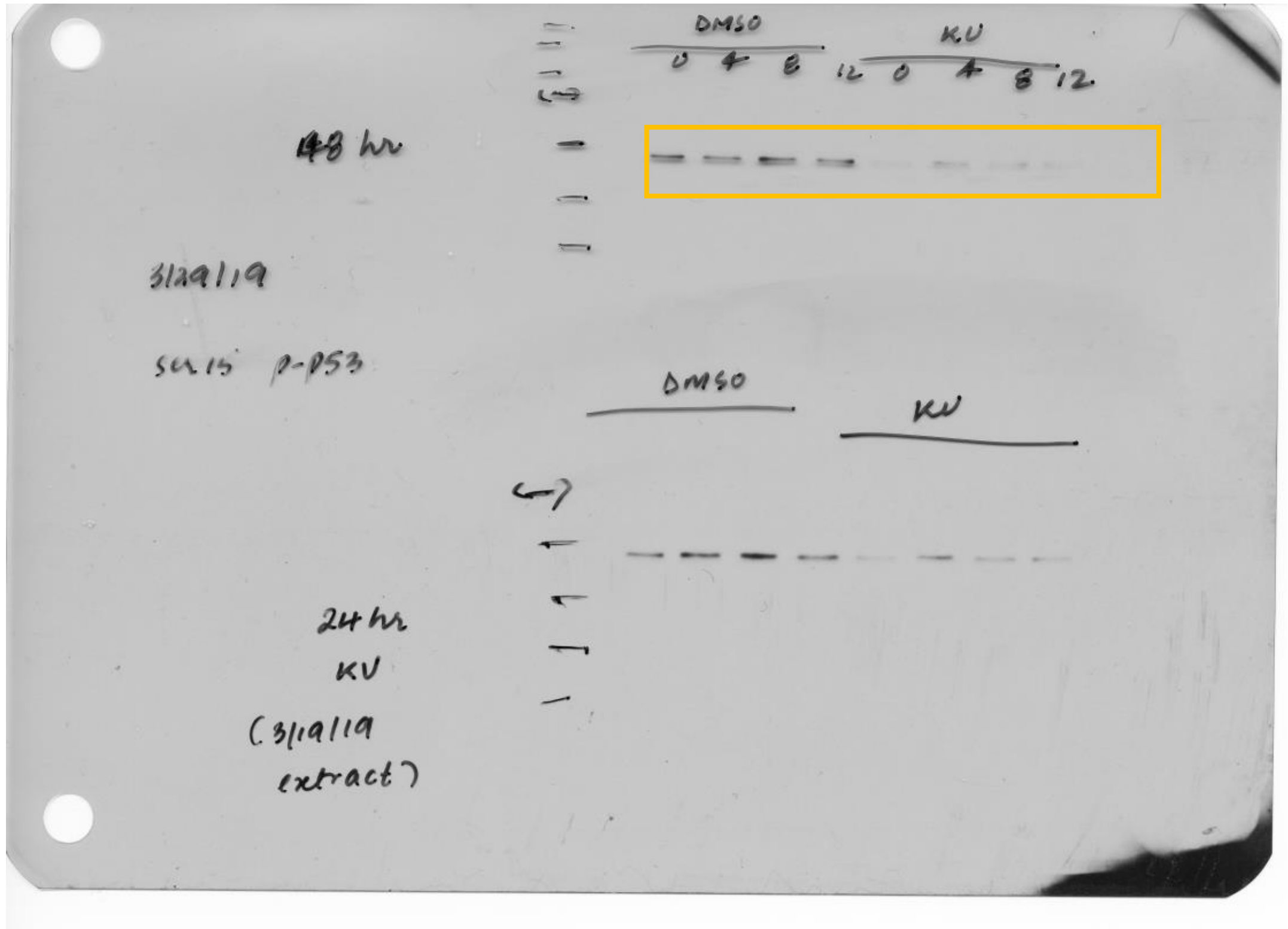
DMSO
KU
0 4 8 12 0 4 8 12
48 hr
3/29/19
ser15 p-p53
DMSO
KU
24 hr
KU
(3/19/19
extract)

Actin

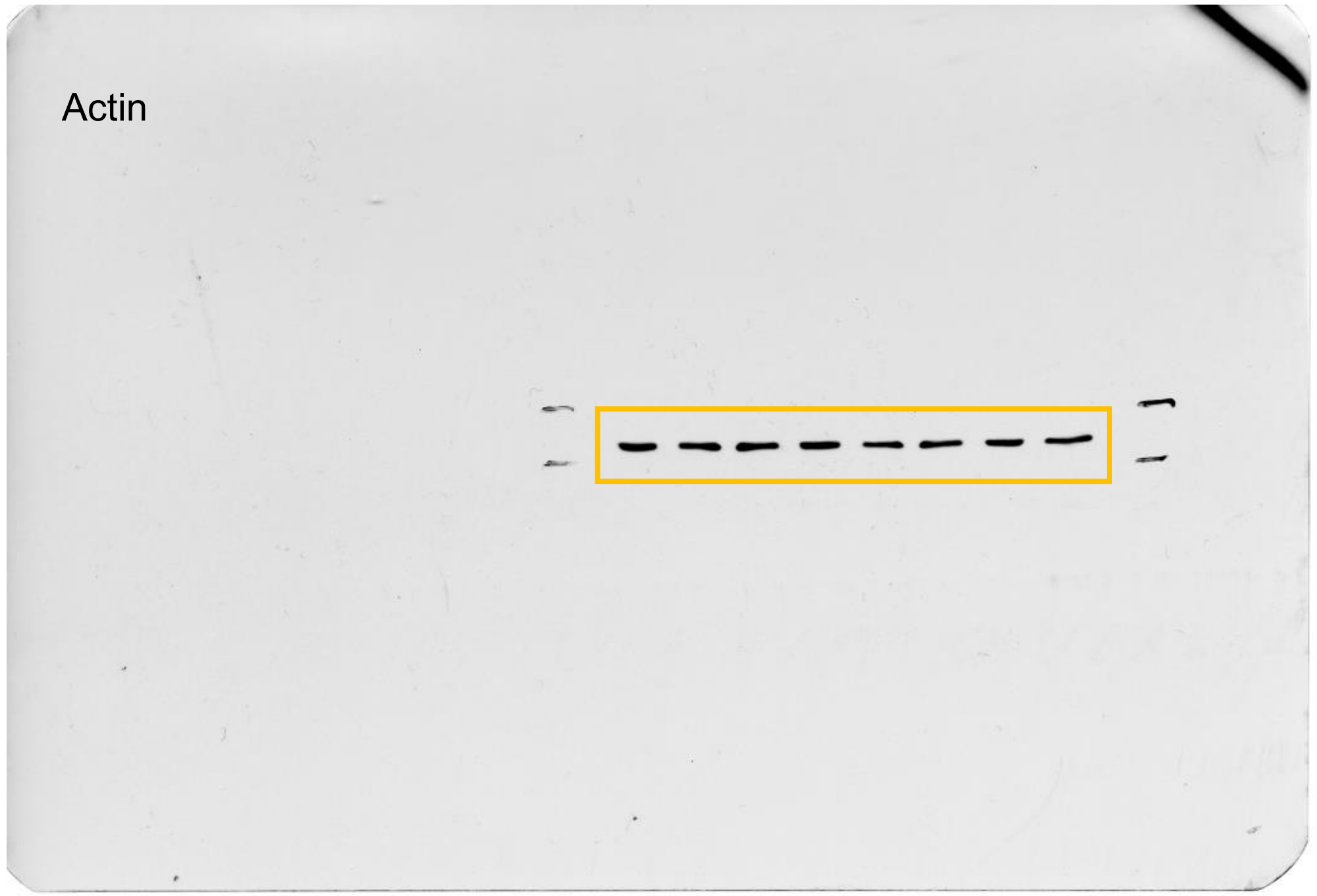

Fig. 6C

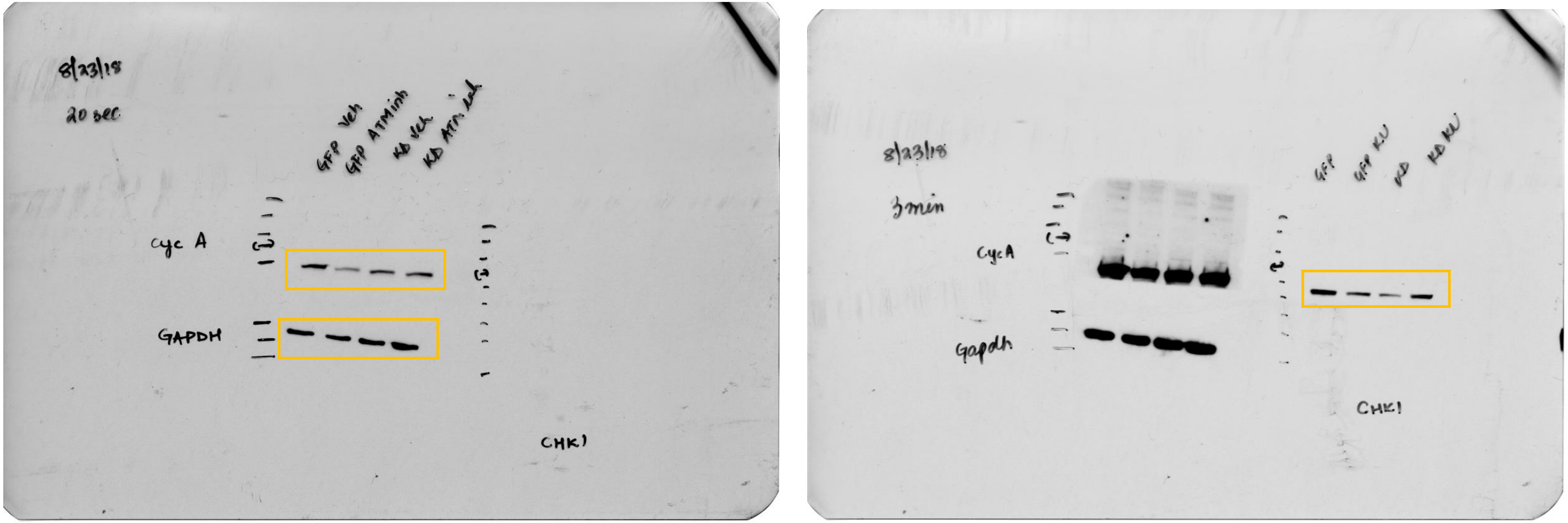

8/23/18
20 sec
GFP Veh
GFP ATMinh
KD Veh
KD ATM inh
cyc A
GAPDH
CHK1
8/23/18
3min
CycA
Gapdh
GFP
GFP KU
KD
KD KU
CHK1

Fig. S1A

G1:shGFP, P1:shp53

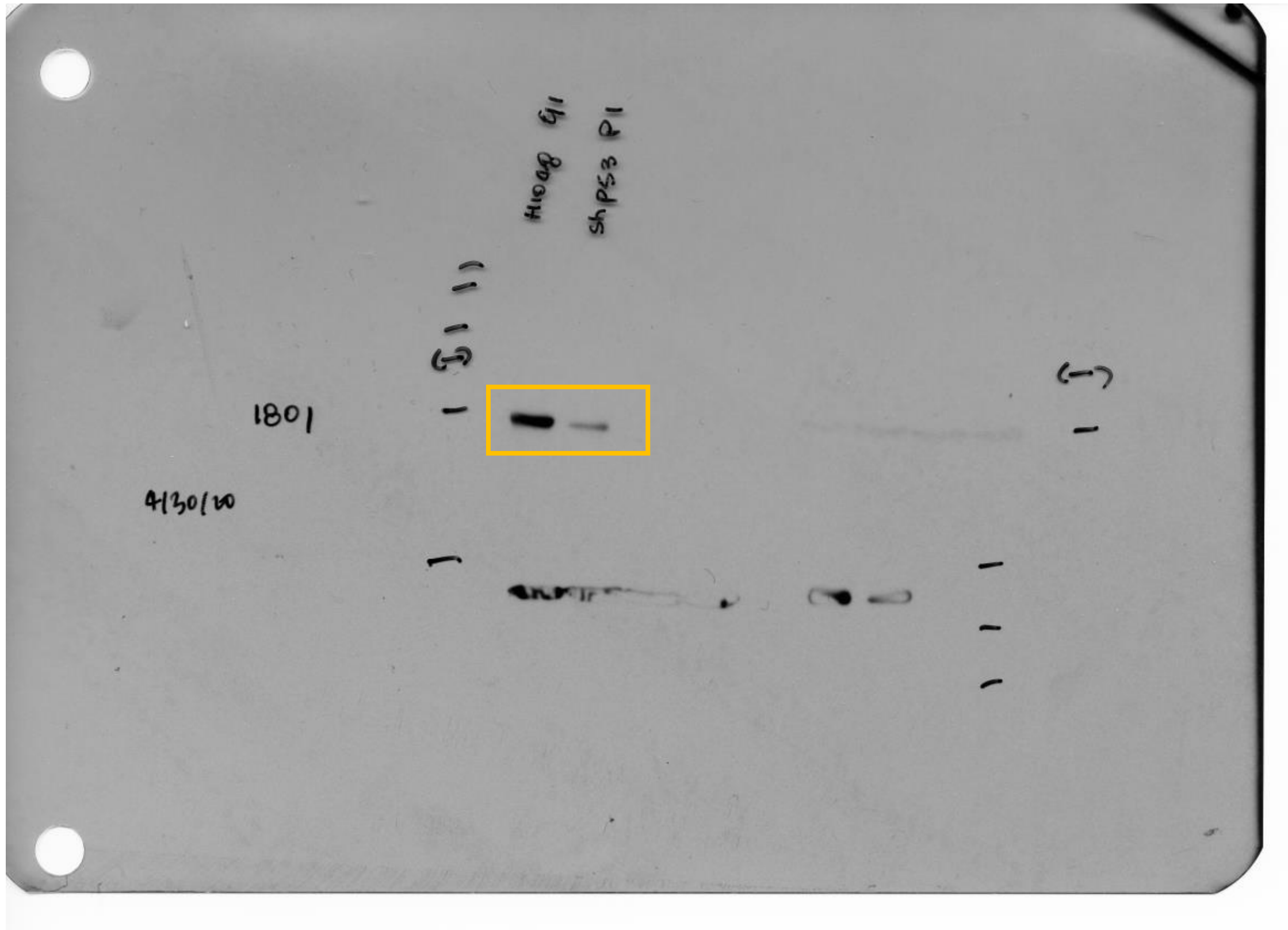

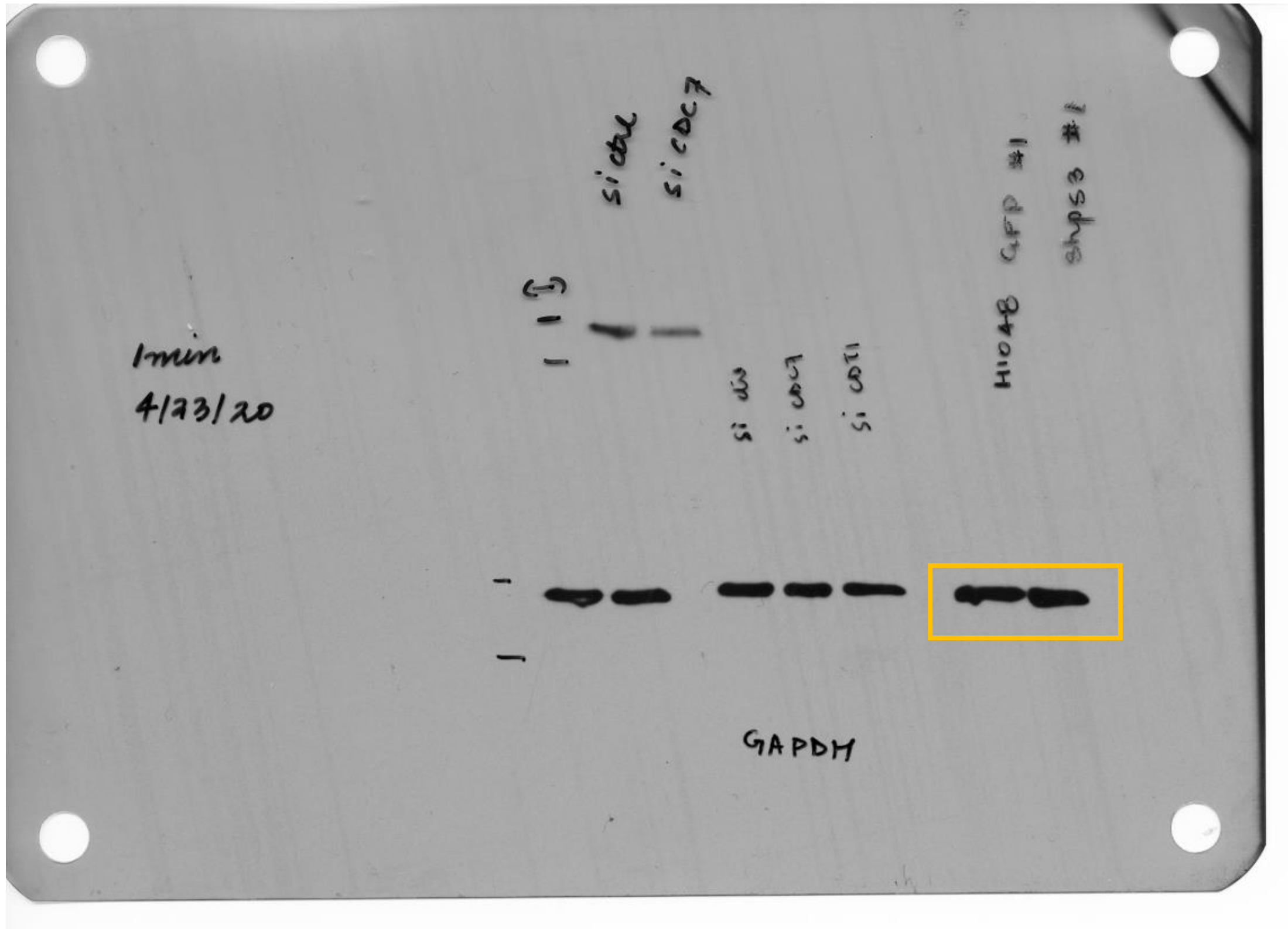

Fig S1G

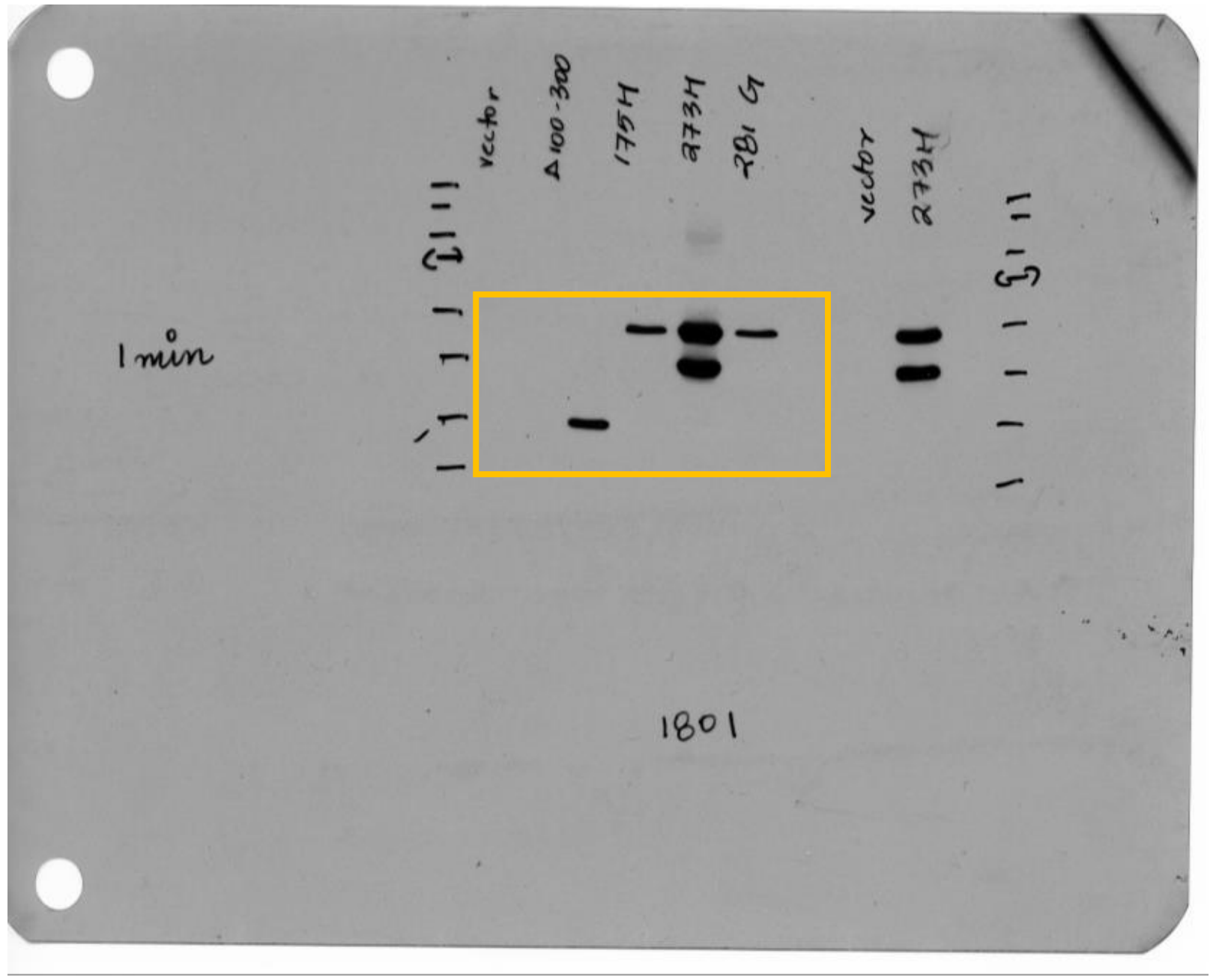
vector
Δ100-300
175H
273H
281G
vector
273H
1 min
1801

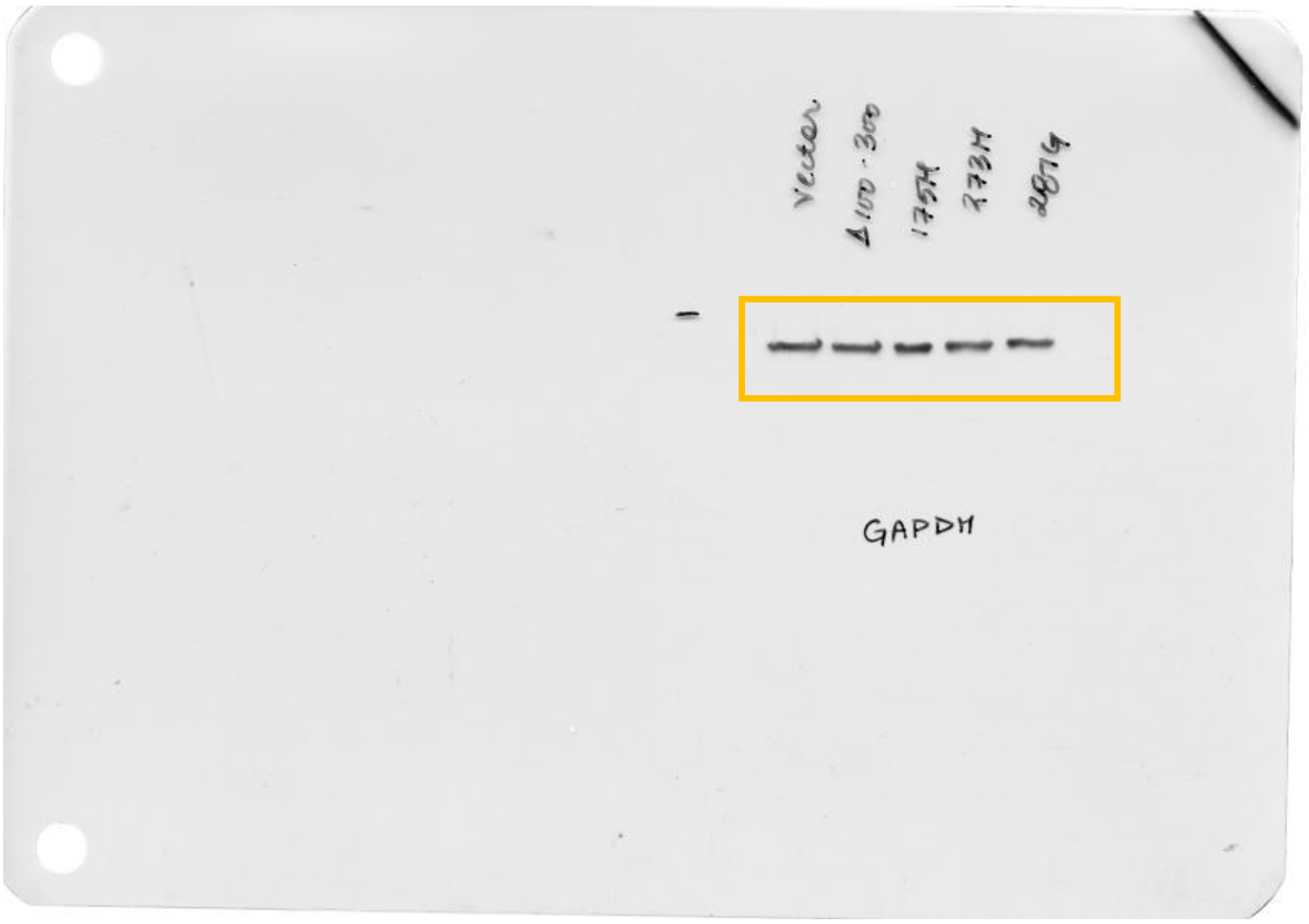
vector
Δ100-300
175H
273H
281G
GAPDH

Fig. S2G

Two clones each of shGFP (G lanes) and shp53 (indicated either as K for knockdown or P for shp53) were used.

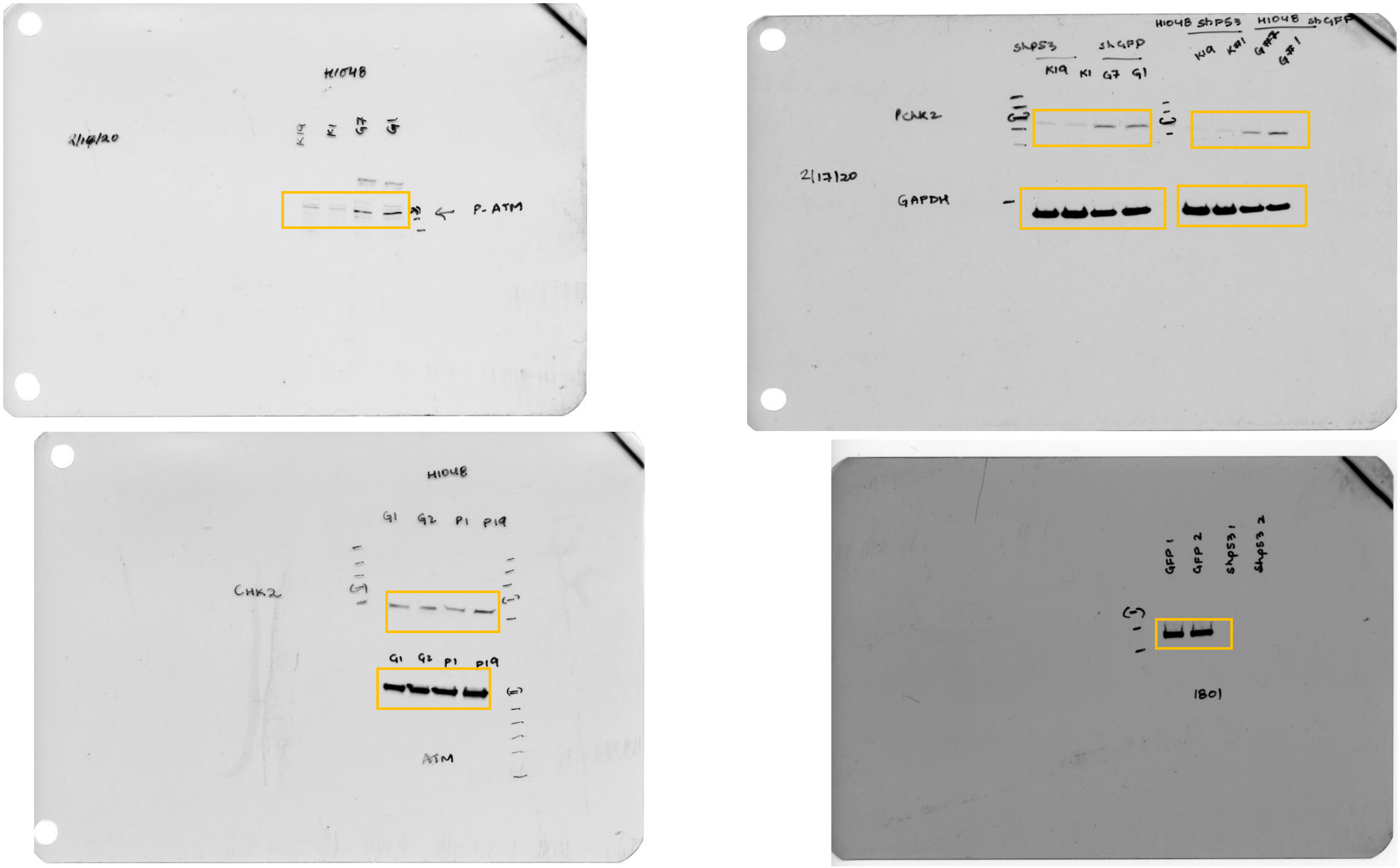

Fig. S2H

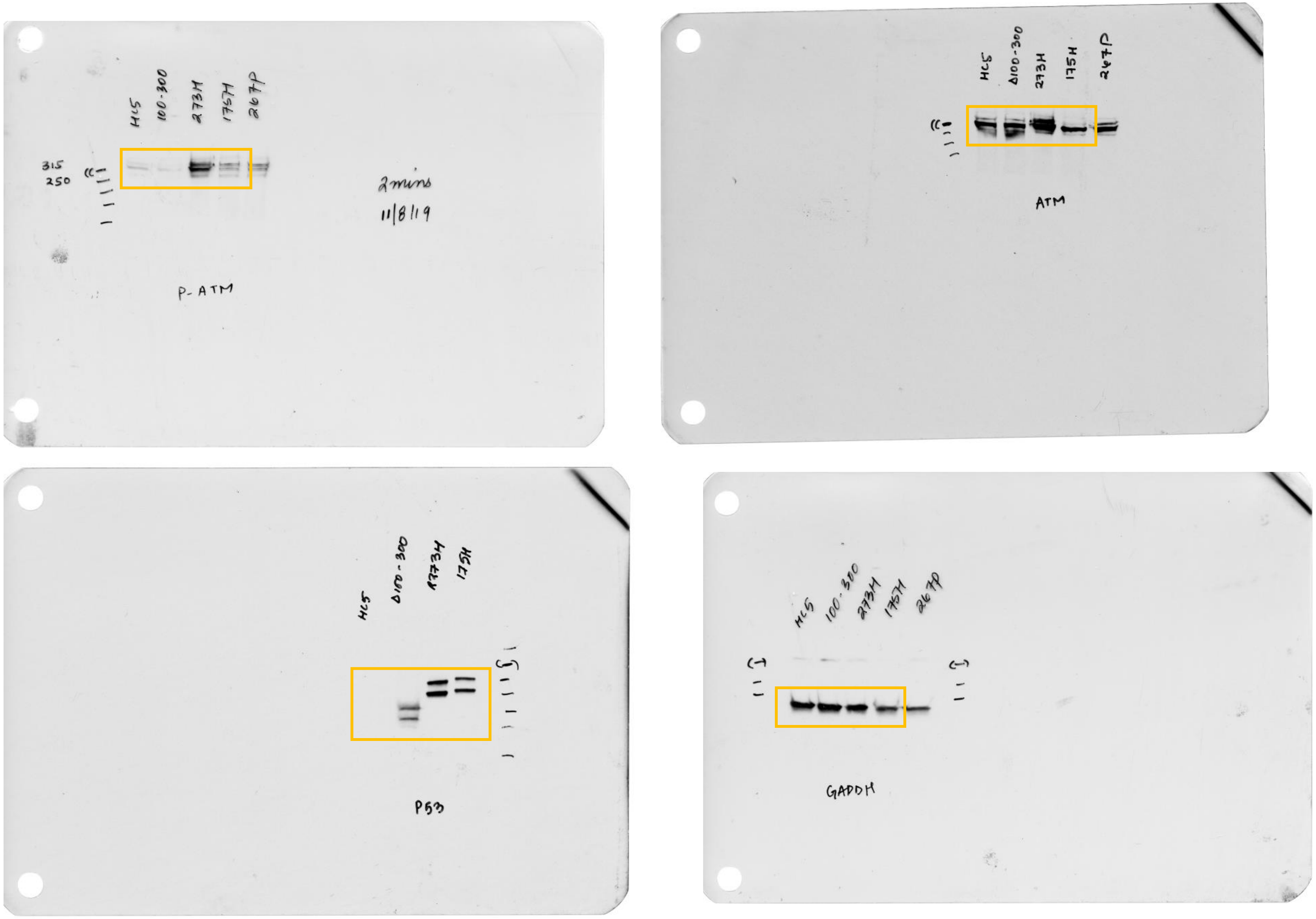
HCS
100-300
273H
175H
267P
315
250
2mins
11/8/19
P-ATM
HCS
Δ100-300
273H
175H
267P
ATM
HCS
Δ100-300
R273H
175H
P53
HCS
100-300
273H
175H
267P
GAPDH

Fig. S3

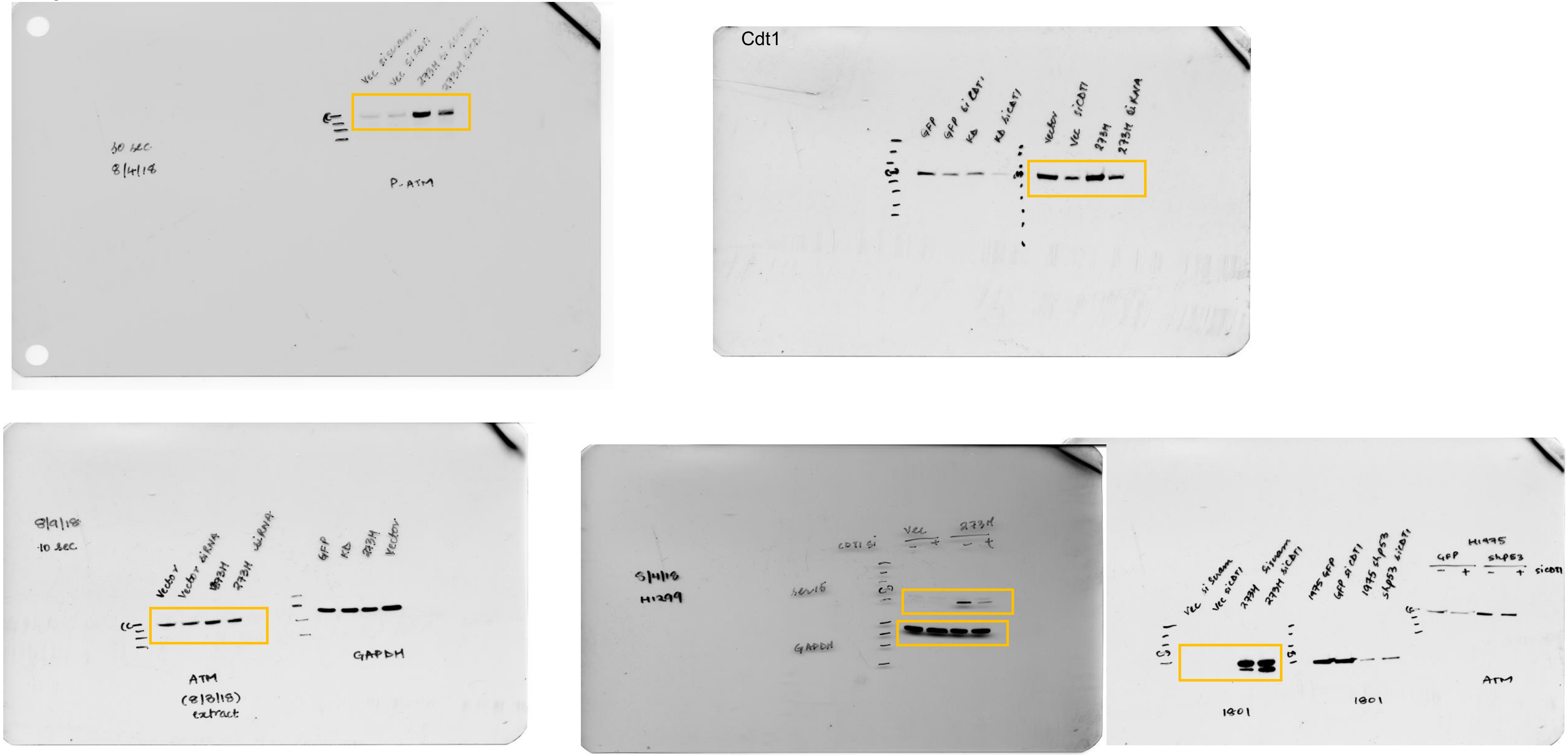

Fig. S6

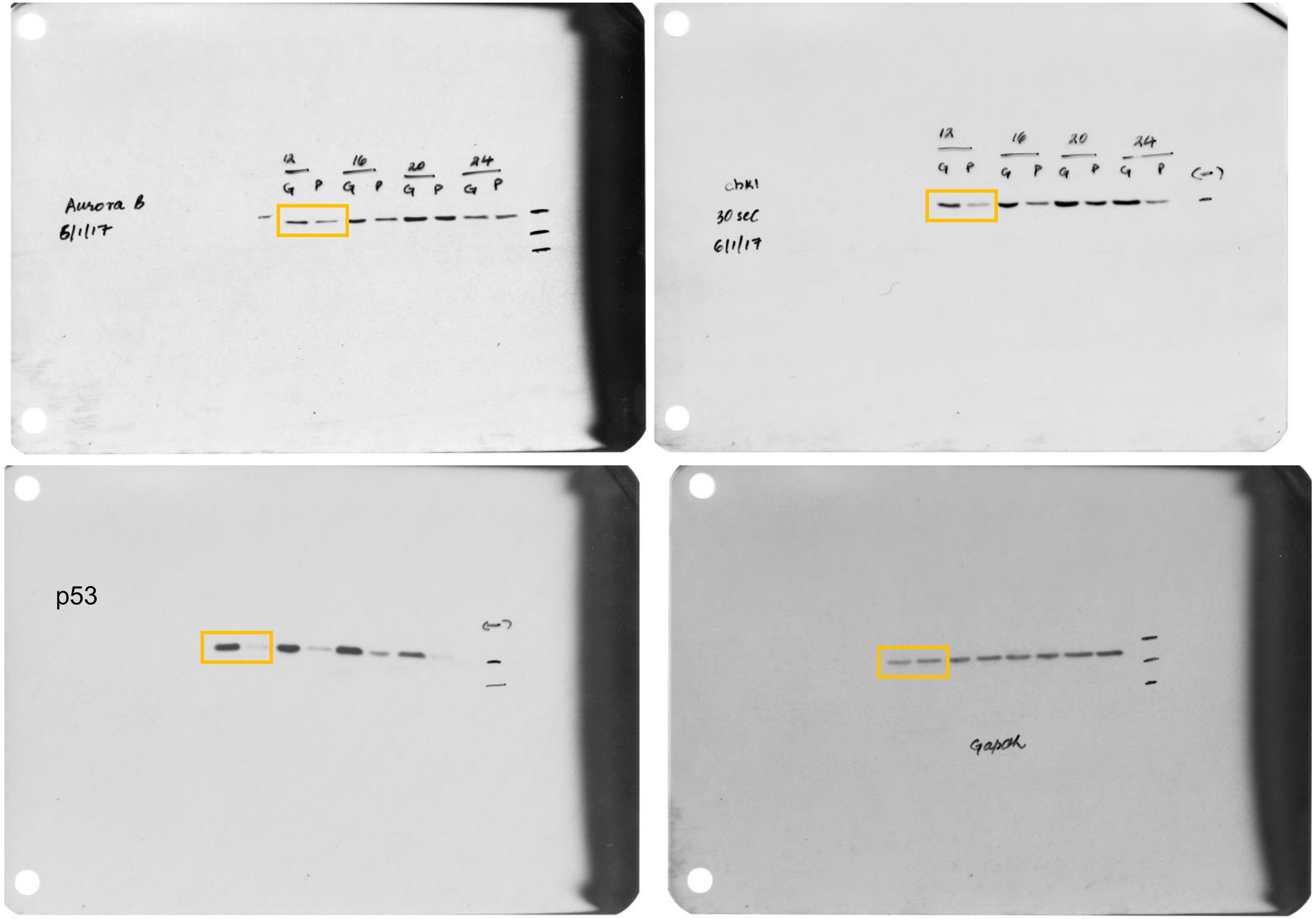
Aurora B
6/1/17
12
16
20
24
G P G P G P G P
chk1
30 sec
6/1/17
12
16
20
24
G P G P G P G P
(-)
p53
(-)
Gapdh

Fig. S9

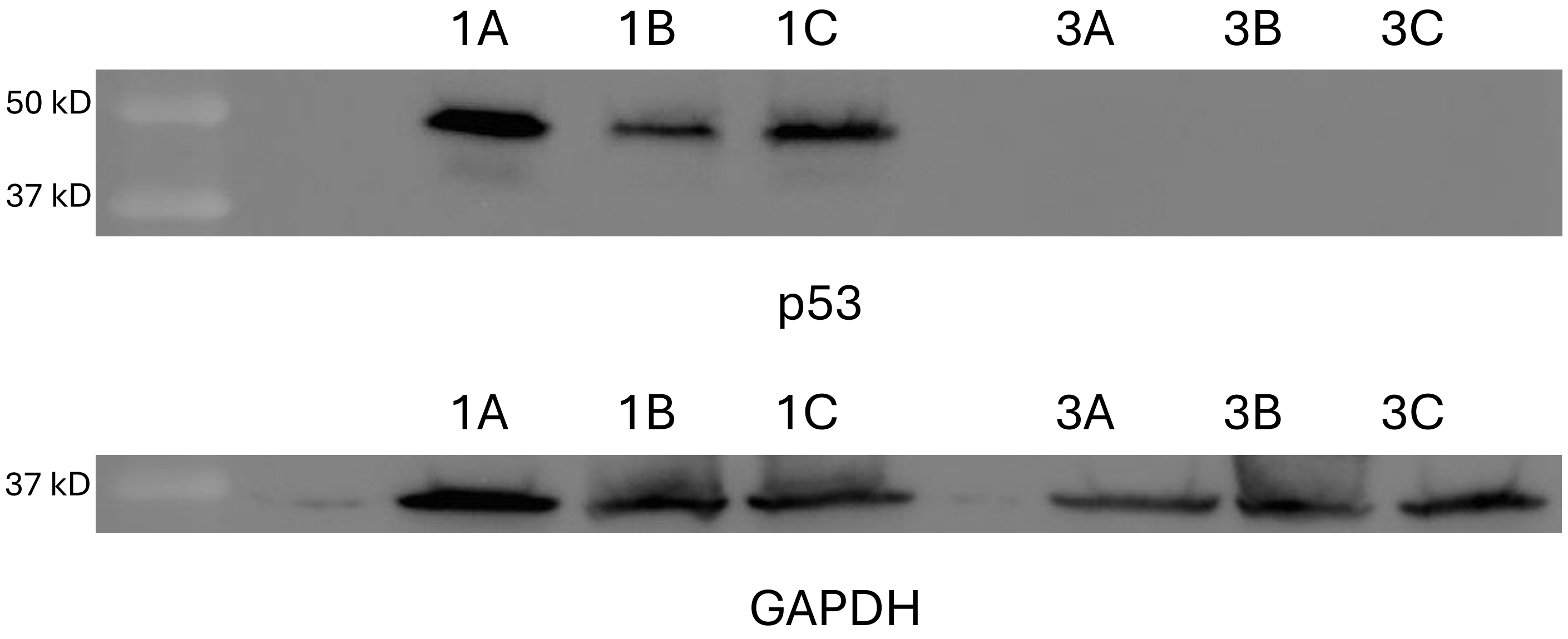

Sample	
H1975 p53 Crispr Control	1A
	1B
	1C
H1975 p53 Crispr KO	3A
	3B
	3C

Sample	
H1975 p53 Crispr Control	1A
	1B
	1C
H1975 p53 Crispr KO +	3A
	3B
	3C
