# Supplementary material for: Oncogenic p53 induces mitotic errors in lung cancer cells by recopying DNA replication forks conferring targetable proliferation advantage
Source: Cell Death Differ. 2026 Jan 26;33(8):1585–97. doi: 10.1038/s41418-026-01670-4 (PMC13433782; doi:10.1038/s41418-026-01670-4)
Supplement: Supplementary file 2 [file 41418_2026_1670_MOESM1_ESM.docx]

**Table S1. Misaligned chromosomes, nuclei associated micronuclei, and nuclear size counted in the PDXs**
