# Supplementary material for: Oncogenic p53 induces mitotic errors in lung cancer cells by recopying DNA replication forks conferring targetable proliferation advantage
Source: Cell Death Differ. 2026 Jan 26;33(8):1585–97. doi: 10.1038/s41418-026-01670-4 (PMC13433782; doi:10.1038/s41418-026-01670-4)
Supplement: Supplementary file 3 [file 41418_2026_1670_MOESM2_ESM.docx]

**Fig. S1. Depletion of endogenous p53-R273C in H1048 human lung cancer cells increases whereas ectopic expression of onc-p53 in p53-null H1299 human lung cancer cells reduces track length of replicating DNA fibers, RPA foci formation and the frequency of re-copied fibers during fork progression,** Upper panel shows (**A**) Onc-p53 levels determined by Immunoblot analysis, (**B**) comparison of track lengths of replicating DNA fibers undergoing elongation in early S phase using Swarm plot analysis, (**C**) Representative confocal images (Magnification 40x) of nuclei with RPA foci, bar graphs comparing (**D**) average number of RPA foci per nuclei, (**E**) percent of nuclei with more than 5 foci and (**F**), percent of re-copied replication forks in mock-depleted (shGFP) and p53-depleted (shp53) H1048 lung cancer cells. Lower panel shows (**G**) expression of indicated Onc-p53 mutants by immunoblot analysis, (**H**) comparison of track lengths of replicating DNA fibers undergoing elongation in early S phase using Swarm plot analysis, (**I**) Representative confocal images (Magnification 40x) of nuclei with RPA foci, (**J**) bar graphs comparing average number of RPA foci per nuclei and (**K**) percent of re-copied replication forks in p53-null H1299 cells stably transfected with vector vs stably expressing the indicated mutants. The percentage of re-copied forks indicated represents percentage of elongating green fibers copying once replicated red fibers (yellow fibers) in a single S phase. p values are shown.

**Fig. S2. Depletion of endogenous p53-R273C in H1048 cells reduces, whereas ectopic expression of Onc-p53 in p53-null H1299 cells increases co-localized 53BP1/H2AX foci formation in G1 nuclei and ATM phosphorylation.** (**A**) Identification of 53BP1 foci in G1 nuclei. Representative confocal images of shGFP and shp53 H1975 nuclei**.** Cells were immunostained with anti-53BP1 (Alexa 594), anti-Cyclin A (Alexa 488) and stained with DAPI (blue). Red arrows show Cyclin A negative G1 nuclei with 53BP1 foci. Scale, 10 µm. Swarm plot (right panel) compares number of 53BP1 foci in G1 shGFP and shp53 H1975 nuclei. 60 cells analyzed in each set. p-value is shown.  **(B)** Representative nuclei from shGFP or shp53 H1048 cells with co- localized 53BP1(red) and γH2AX (green) foci (Magnification 40x) in merged, red and green channels. Bar graphs compare (**C**) average number of co-localized 53BP1 and γH2AX foci per nuclei, and (**D**) percent of nuclei with more than 10 co-localized 53BP1 and γH2AX foci in shGFP or shp53 H1048 cells. (**E**) Representative nuclei with co-localized 53BP1 (red) and γH2AX (green) foci in p53-null H1299 cells stably transfected with empty vector (vector) or stably expressing indicated Onc-p53 (Magnification 40x) in merged, red and green channels. **(F)** Bar graphs compare average number of co-localized 53BP1 and γH2AX foci per nuclei. Approximately 60 nuclei were counted in each foci assay. p-values are shown. Phospho (Ser1981)-ATM (p-ATM) and/or phosphor (Thr68)-Chk2 (p-Chk2) levels in **(G)** shGFP or shp53 H1048 cells, **(H)** H1299 cells stably transfected with empty vector (vector) or stably expressing indicated Onc-p53 mutants. Comparative p-ATM or p-Chk2 levels in the immunoblots were determined by densitometry and normalized by loading control GAPDH are indicated.

**Fig. S3. Partial depletion of Cdt1 in H1299 cells stably expressing p53-R273H downregulates ATM signaling.** Immunoblot analysis to detect phosphor (Ser1981)-ATM (p-ATM) and phosphor (Ser15)-p53 (p-p53) levels in H1299 cells stably transfected with empty vector (Vector) or stably expressing p53-R273H (R273H) after transfection with sicontrol (-) or siCdt1 (+) RNA. Comparative levels of p-ATM and p-p53 determined by densitometry and normalized by loading control GAPDH are indicated at the bottom

**Fig. S4. Mitotic segregation errors in human lung cancer cells and tumors. during serial passages.** (**A**) Representative images of shCT and shp53 H1975 nuclei immunostained with anti-p53 (Alexa 594) and stained with DAPI (blue) showing micronuclei (white arrow) and multi-nucleated cells (yellow arrow). Two shControl and shp53 RNA expressing cells were used (**B**) Representative image of H&E stained FFPE sections of patient-derived NSCLC tumors with WT or mutant (R158L and R159P) p53 alleles. Images show mitotic features in primary (P1) and serially passaged (P3) tumor cells. Scale, 10 µm.

**Fig. S5. Inhibition of ATM signaling drastically reduces CCNA2 and CHEK1 expression**. Expression of (**A**) CCNA2 and (**B**) CHEK1 transcript levels normalized with GAPDH in p53-null H1299 cells stably transfected with empty vector (Vector) or stably expressing p53-R273H (R273H) at the early S phase in the presence of vehicle (DMSO) or ATM inhibitor, KU-600019, (ATMi).

**Fig. S6. Small molecule inhibitors of AURKB, and combined treatment with Chk1 and ATM inhibitors synergistically and preferentially inhibit proliferation of H1975 (Onc-p53 R273H) lung cancer cells, while Chk1 and ATM inhibitors mildly delay mitotic entry and growth rate of normal lung fibroblast WI38 cells not inducing cell death.** Onc-p53 upregulate expression of AURKB conferring dependency in H1975 cells (**A-C**). Expression of Chk1, p53 and AuroraKB in H1975 shGFP or shp53 cells. GAPDH is a loading control (**A**). Treatment with AuroraKB inhibitor (Barasertib, 0.5µM) prevented cytokinesis accumulating multinuclear cells only in mock-depleted (shGFP) H1975 cells (**B**) and preferentially blocking cell growth (**C**). Growth rate of H1975 (**D**) shGFP or (**E**) shp53 cells treated with vehicle (DMSO) or 2 µM Chk1 inhibitor, MK8776 (Chk1i,) and 3 µM ATM inhibitor, KU-600019 (ATMi), individually or in combination. Error bars indicate +/- 1 S.D. (**F**) Synergistic inhibition of H1975 shGFP cells by Chk1i and ATMi (combination index 0.2) determined from percent survival of cells after 72 hours at indicated drug concentrations. **(G)** Rate of asynchronous W138, normal lung fibroblast cells entering mitosis over time were recorded by phase-contrast video-microscopy at one image/5 min in the indicated conditions. Horizontal bar graphs represent the rate of asynchronous WI38 cells from interphase (grey bars) to mitosis (blue bars) over time, acquired from live time lapse imaging in the presence of DMSO, or 2 µM Chk1i, and/or 3 µM ATM inhibitor, KU-600019 (ATMi). Entry into mitosis for each cell was defined as nuclear envelop break down. **(H)** Growth rate of W138 cells in the presence of DMSO, or 2 µM Chk1i, and/or 3 µM ATMi) Error bars are +/- 1 S.D.

**Fig. S7.** **Small molecule inhibitors of Chk1 and ATM do not inhibit proliferation of xenograft tumors from H460 (Wtp53), but cooperate with each other to preferentially inhibit proliferation of xenograft tumors from H1975 (Onc-p53 R273H) lung cancer cells.** (**A**) Rate of Proliferation of H460 (WT 53) xenograft tumors in the presence of vehicle (Veh), or MK8776 (Chk1i), 50mg per Kg twice a week and AZD0156 (ATMi), 10 mg per Kg oral daily individually or in combination as indicated. (**B**) Orthotopic tumor growth of H1975 cells expressing luciferase in lung of immunodeficient mice in the presence of vehicle or Chk1i+ATMi. Representative bioluminescence images treated from 7 to 21 days using oral or nasal delivery in 5 mice per condition. Representative ex-vivo bioluminescence images of lung and liver (Bottom panel). Swarm plot shows total luminescence flux at the endpoint.

**Fig. S8. A small molecule inhibitor of Chk2, a downstream target of ATM, cooperates with a Chk1 inhibitor to preferentially inhibit proliferation of shGFP H1975 lung cancer cells.** Growth rate of H1975 cells stably expressing (A) shGFP or (B) shp53 in the presence of vehicle (DMSO) or 2 µM Chk1 inhibitor, MK8776 (Chk1i) or 5 µM Chk2 inhibitor, Chk2 inhibitor II hydrate (Chk2i,) individually or in combination. Experiments were repeated three times. Error bars indicate +/- S.D. (C). Synergistic inhibition combination index (0.5) of shGFP H1975 cells by inhibitors of Chk1 (Chk1i) and Chk2 (Chk2i) determined from percent survival of cells at indicated drug concentration. (**D**, **E**) Swarm plots of xenograft tumor weights from (**D**) shGFP and (**E**) shp53 H1975 cells after indicated drug treatments. Palpable tumors were treated for 2 weeks with vehicle (-) or MK8776 (Chk1i), 50mg per Kg twice a week and Chk2 inhibitor II hydrate (Chk2i) 5mg per Kg oral daily individually or in combination as indicated. Seven mice (two tumors/mouse) were used in each group. p values are shown. (**F, G**) TUNEL assay for apoptosis in tumors from shGFP and shp53 H1975 lung cancer xenografts treated with Chk1 and Chk2 inhibitors. (**F**) Representative pictures and (**G**) Swarm plot of vehicle (-), Chk1 (Chk1i) and/or Chk2 (Chk2i) inhibitor-treated (two consecutive treatments at 24 hours interval) tumor sections generated from shGFP and shp53 H1975 xenografts show apoptotic (green fluorescence) cells by TUNEL assay. p values are shown.

**Fig. S9. Crispr Knockout of Onc-p53 R273H in H1975 cells abolishes its tumor formation ability. (A)** Swarm plots of tumor weight (gm) and **(B)** growth rate of xenograft tumors from H1975 p53-Crispr control and Crispr KO cells (1.5X10^6^) are shown. Tumor weight and cumulative tumor volume (mm^3^) developed in each mouse at the study end point was determined. Five mice with tumors on both sides were used in each group. p values for both A (Mann Whitney Wilcoxon) and for B (two-way Annova) is 0.0007. **(C)** Immunoblot analysis using chemiluminescence imaging to demonstrate p53 expression in H1975 p53-crispr control and Crispr KO cells. GAPDH is loading control.
